# Brain areas associated with numbers and calculations in children: Meta-analyses of fMRI studies

**DOI:** 10.1016/j.dcn.2017.08.002

**Published:** 2017-08-08

**Authors:** Marie Arsalidou, Matthew Pawliw-Levac, Mahsa Sadeghi, Juan Pascual-Leone

**Affiliations:** a Department of Psychology, Faculty of Health, York University, Toronto, Canada; b Department of Psychology, National Research University Higher School of Economics, Moscow, Russian Federation

**Keywords:** Mathematical cognition, Meta-analyses, fMRI, Children, Development, Insula

## Abstract

Children use numbers every day and typically receive formal mathematical training from an early age, as it is a main subject in school curricula. Despite an increase in children neuroimaging studies, a comprehensive neuropsychological model of mathematical functions in children is lacking. Using quantitative meta-analyses of functional magnetic resonance imaging (fMRI) studies, we identify concordant brain areas across articles that adhere to a set of selection criteria (e.g., whole-brain analysis, coordinate reports) and report brain activity to tasks that involve processing symbolic and non-symbolic numbers with and without formal mathematical operations, which we called respectively number tasks and calculation tasks. We present data on children 14 years and younger, who solved these tasks. Results show activity in parietal (e.g., inferior parietal lobule and precuneus) and frontal (e.g., superior and medial frontal gyri) cortices, core areas related to mental-arithmetic, as well as brain regions such as the insula and claustrum, which are not typically discussed as part of mathematical problem solving models. We propose a topographical atlas of mathematical processes in children, discuss findings within a developmental constructivist theoretical model, and suggest practical methodological considerations for future studies.

From an early age we learn to live in a world with numbers: on classroom doors, street signs, price tags, our phones, on our work activities. Typically we learn how numbers and quantities relate to each other (e.g., smaller, larger) from an early age; and most of us received some formal training in math, starting from grade school. Grade school training in mathematics coincides with protracted development of the pre-frontal cortex (e.g., Gogtay et al., 2004). The pre-frontal cortex is a key brain region, concordant across mathematical-cognition studies in healthy adults (Arsalidou and Taylor, 2011, for meta-analyses). Much progress has been made in understanding brain correlates of mathematical cognition; however, despite the increase in the studies examining children’s mathematical problem solving (i.e., quantity discrimination and mathematical operations), a neuropsychological model for children is still not available. We have compiled data from functional magnetic resonance imaging (fMRI) studies and report concordant findings on brain correlates of typically developing children when solving math tasks with and without formal calculations (i.e., operations).

Behavioral protocols with children can be designed using math tasks such as printing or naming numbers, counting and sorting objects (Agostino et al., 2010, LeFevre et al., 2009). Neuroimaging tasks, however, are largely restricted to the visual domain, because they need to adhere to constraints/limitations of the imaging methodology (Kotsoni et al., 2006, Arsalidou and Im-Bolter, 2016, Arsalidou and Pascual-Leone, 2016). For fMRI studies, task protocols must be as time limited as possible. For instance, stimulus presentation should be brief, a few seconds; longer intervals are harder to control for irrelevant intruding processes (e.g., mind wandering). Manual responses are preferable to verbal responses, being less likely to produce head motion that compromise image quality. Moreover, calculation tasks are typically simple, often 1- or 2- digit operations, so that participants can provide a response within a limited time frame. Most fMRI studies that examine brain correlates of mathematical cognition, either in children or adults, follow these basic task characteristics.

The majority of fMRI studies in the literature investigated mathematical cognition in adults (e.g., Menon et al., 2000, Fehr et al., 2007), and the parietal lobes received the most attention in early studies of mental arithmetic. Indeed, parietal brain regions, such as bilateral intraparietal sulci, left angular gyrus, and bilateral superior parietal cortices, play distinct roles in number processing (Dehaene et al., 2003). Although the parietal cortex is fundamental to process mathematical problems, other regions are involved as well (Ansari et al., 2005, Ischebeck et al., 2009, Zago et al., 2008, Zhou et al., 2007). Coordinate-based meta-analyses of 53 adult fMRI studies show areas, such as cingulate gyri, insula and the prefrontal cortex that are concordantly active in tasks of numbers and calculation (Arsalidou and Taylor, 2011).

*Number tasks* are those that contain numbers (e.g., viewing different digits) and quantities (e.g., viewing small or large arrays of dots), but require no formal calculation (e.g., multiplication). They have in common some sort of semantic judgement on numbers or quantities based on stipulated rules. *Calculation tasks* require as well operation rules, such as addition, subtraction and multiplication, applied to numbers. Distinct and common brain areas are active in adults during number and calculation tasks (Arsalidou and Taylor, 2011). Specifically, they elicit brain responses within regions such as inferior parietal lobule and inferior frontal cortex; however, calculations also recruit prefrontal areas, particularly in middle and superior frontal gyri (Arsalidou and Taylor, 2011).

The first fMRI study with children was published 16 years ago, by Eliez et al. (2001), testing children with or without a velocardiofacial syndrome, on an arithmetic task. fMRI studies of children working on mathematical problems are gradually increasing. As in adult studies, tasks administered to children divide into those investigating numerical processes (e.g., Ansari et al., 2005, Ansari and Dhital, 2006, Cantlon et al., 2006, Klien et al., 2014) and those studying mathematical operations (e.g., Ashkenazi et al., 2012, Davis et al., 2009a, Du et al., 2013, Metcalfe et al., 2013).

Studies that examine numerical processes typically ask children to select the larger number in a set of numbers (e.g., Ansari et al., 2005). The numbers in the set can differ by either small differences/distance (i.e., 1, 2 and 3) or large (i.e., 5, 6, and 7) ones. When the difference is small, children show activity in the superior parietal lobe, medial and inferior frontal gyri, the insula, and subcortical regions − mostly in the right hemisphere (Ansari et al., 2005). A subsequent study by the same researchers shows that when number differences are large, children activate the left hemisphere’s dorsolateral prefrontal cortex, inferior frontal gyrus, and intraparietal sulcus (Ansari and Dhital, 2006). Other studies compared brain responses to numbers versus responses to shapes (Cantlon et al., 2006); or examined numerical processes versus a control task (Kaufmann et al., 2008).

Procedural differences in mathematical operations often lead to activity in different cortical regions (Kawashima et al., 2004, Prado et al., 2014). Studies examining various operations in the same children are important: Prado et al. (2014) examined brain responses to subtraction and multiplication, and Kawashima et al. (2004) examined three mathematical operations, addition, subtraction and multiplication. They found several common brain regions associated with them all. In the prefrontal cortex, for instance, addition and subtraction recruit the left middle frontal cortex, whereas multiplication recruits left middle and inferior frontal cortices (Kawashima et al., 2004); further, unlike addition and multiplication, subtraction elicited activity in the right intraparietal sulcus. More fMRI studies are needed that examine multiple mathematical operations in the same children.

A meta-analysis by Houdé et al. (2010) reports concordance across seven fMRI studies, which tested children with either number or calculation tasks, in right inferior and middle frontal gyri, left superior frontal gyrus and left middle occipital gyrus. This meta-analysis supports the view that prefrontal regions play an important role in mathematical cognition (Rivera et al., 2005, Ansari, 2008, Arsalidou and Taylor, 2011). Houdé et al. (2010) did not detect extensive involvement of parietal cortex, which is critical in mathematical cognition, possibly because of variability in the original studies’ methodology and the low number of foci. Another meta-analysis examined 19 fMRI studies that included children (Kaufmann et al., 2011). Perhaps due to the low number of studies, the authors (Kaufmann et al., 2011) chose to include studies with fixed effects analyses (e.g. Kaufmann et al., 2006, Chen et al., 2006), coordinates from contrasts with variable performance (i.e., interaction of brain activity of high and low performers, Kovas et al., 2009), and variable age (i.e., coordinates resulting from a conjunction analysis between children and adults, Holloway and Ansari, 2010); they also included age ranges spanning over participants older than 18 years (i.e., 8.53–19.03 years, Rivera et al., 2005). Although such approach increases the number of studies and coordinates in the meta-analyses, it obscures the reliability of results.

Targeted meta-analyses were recently performed to identify brain correlates of number processing and notation (i.e., symbolic vs non-symbolic) in adults (Sokolowski et al., 2017) and children (Kersey and Cantlon, 2017). These studies suggest a network of parietal and frontal areas that underlie symbolic and non-symbolic processes. Based mainly on adult data theoretical models of mathematical cognition (e.g., Dehaene and Cohen, 1997, Arsalidou and Taylor, 2011) may not be suitable for accounting for developmental data (Arsalidou and Pascual-Leone, 2016). Also, it is challenging to identify developmental theories of cognition that make clear neural predictions on mathematical processes. We used a domain general cognitive theory of development for hypotheses building. The Theory of Constructive Operators (Pascual-Leone, 1970, Pascual-Leone and Johnson, 2005, Arsalidou and Pascual-Leone, 2016) outlines brain correlates associated with schemes and operation types to predict performance. The theory of constructive operators would predict that brain responses to number and calculations tasks are not material-driven, but process-driven and vary with the trade-off between participants’ mental-attentional capacity and the mental demand of the task. Specifically, this trade-off predicts that the right hemisphere is involved in processing of automatized schemes, whereas the left hemisphere is involved in processing problems that involve the child’s mental-attentional capacity, and are not automatized yet (details on this account is given in the discussion). Thus, we anticipated that number tasks should favour right frontal and parietal regions, whereas calculation tasks within the child’s mental-attentional capacity will recruit additional frontal and parietal regions in the left hemisphere. In the current meta-analyses we explore brain areas involved in mathematical cognition of children younger than 14 years, and provide normative fMRI atlases in standard stereotaxic space for number and calculation tasks.

## 1 Methods

### 1.1 Literature search and article selection criteria

**Fig. 1:**
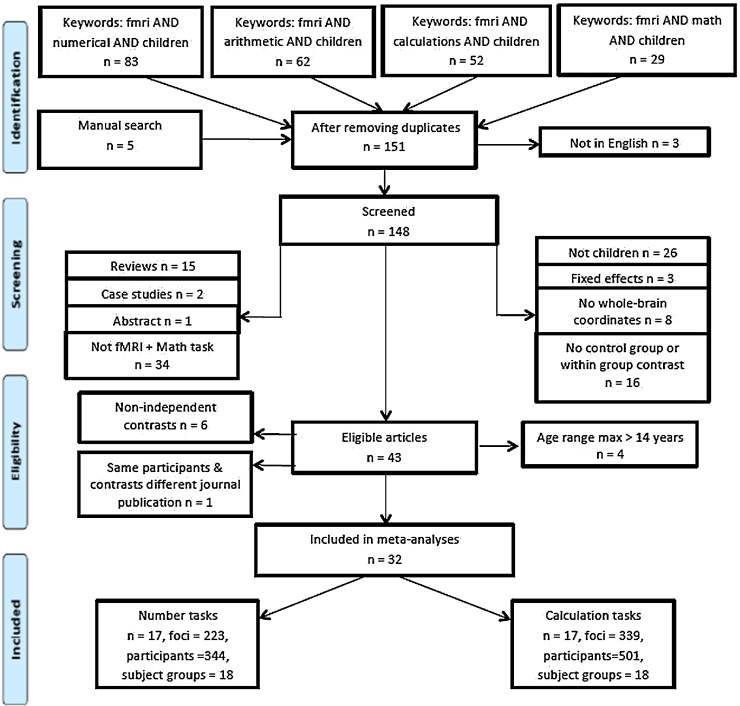
PRISMA flowchart for identification and eligibility of articles (template by Moher et al., 2009). n = number of papers.

The literature was searched, in June 2017, by means of web-of-science (http://www.isiknowledge.com), using the terms fMRI and arithmetic and children; fMRI and calculations and children; fMRI and math and children; and fMRI and numerical and children. We have also added five papers using manual search. Fig. 1 shows the number of articles from this search, and the process we followed to identify eligible articles. Specifically, after eliminating duplicates, the articles were subjected to a series of selection criteria. For inclusion articles needed to: (a) be written in English, (b) have used fMRI and tasks involving numbers and mathematical operations; (c) have healthy children participants as the main or control group; (d) have reported whole-brain, within-group results using random-effects analysis; (e) have reported stereotaxic coordinates in Talairach or Montreal Neurological Institute (MNI) coordinates. Forty-three articles survived these criteria. To maintain data independence, we eliminated articles that reported contrast with analyses involving other age groups (e.g., conjunction between children and adults) and/or other tasks (e.g., conjunction between working memory and arithmetic problem solving). We also eliminated articles that included participants over 18 years in the children group (i.e., age range 8.53–19.03 years. Rivera et al., 2005; age range 7.7–21 years, Kesler et al. (2006); mean age 17 years 11.5 months, Price et al., 2013), and one article for including the same experiments (i.e., contrasts) using same participants in different publications (Meintjes et al., 2010a, Meintjes et al., 2010b). These controls resulted in 32 acceptable articles testing children 14 years or younger.

Contrast coordinates were selected based on whether the experimental paradigm was related either to number tasks or calculation tasks. *Number tasks* were defined as those involved in numerical processing without having prescribed operations (e.g., number-distance effects, numerical comparisons). In the number tasks meta-analyses we included data from 18 independent subject-groups that included 30 experiments (i.e., contrasts) using symbolic (i.e., Arabic) stimuli (Ansari et al., 2005, Berteletti et al., 2014, Cantlon et al., 2006, Demir-Lira et al., 2016, Kaufmann et al., 2008, Kucian et al., 2011a), non-symbolic (e.g., dots) stimuli (Ansari and Dhital, 2006, Berteletti et al., 2015, Bugden et al., 2012, Gullick and Wolford, 2013, Kucian et al., 2011b, Libertus et al., 2009, Meintjes et al., 2010b, Vogel et al., 2015), or a combination of both (Emerson and Cantlon, 2012, Park et al., 2014). *Calculation tasks* were defined as tasks that involved prescribed mathematical operations − addition, subtraction or multiplication. Included among calculation tasks were 42 experiments from 18 independent subject groups that used symbolic stimuli, with three exceptions: one paper using non-symbolic stimuli (Ashkenazi et al., 2012) and two others having a combination of symbolic and non-symbolic stimuli (Krinzinger et al., 2011, Peters et al., 2016). Insufficient experiments (less than 17–20 experiments; Eickhoff et al., 2017) did not allow us to examine operations separately. Similarly, due to insufficient data of symbolic and non-symbolic experiments in children under 14 years, we could not investigate the hypothesis of whether numbers have abstract or multiple representations (e.g., Cohen Kadosh and Walsh, 2009). Three experimenters (MA, MPL and MS) performed contrast selection separately, and final decisions were taken in agreement. Table 1 shows article information, participant demographics, and contrast selection for each category.

**Table 1:** Descriptive information of articles and contrasts used in the meta-analyses.

Author	Year	Sample (N)	F	Hand	Age	Number tasks	Foci
Ansari	2005	12	n/r	n/r	10.4 (9.2–11.11)	Distance effect (small > large)	8
Ansari	2006	9	3	R	10.4 (9.11–11.11)	Distance effect (small > large)	3
Berteletti	2014	20	11	n/r	11.5 SD = 1.7 (8.6–13.1)	Numerosity Task	1
Berteletti	2015	39	22	n/r	11:4, SD = 1:6 (8:5–13:7)	Numerical Processing Large vs Small	4
Bugden	2012	17	14	R	105.5 m SD 6.09 m (95–116 m)	Distance effect ratio	5
Cantlon	2006	8	5	n/r	4.75 (4.25–4.95)	Number > shape	7
Demir-Lira	2016	33	20	n/r	10.9 ± 1.5 (8–13.8)	Spatial localizer (dot arrays) at Time 1	4
Emerson	2012	24	n/r	n/r	8.24 SD = 2.26 (4.32–11.86)	Number network	5
Gullick	2013	16	6	R	10y;8 m (9;11–11;9)	Fifth graders: Positive > Negative numbers	11
						Fifth graders: Negative > Positive numbers	1
						Fifth graders: Positive distance effect	9
						Fifth graders: Negative distance effect	19
						Fifth graders: Positive > Negative distance effect	5
						Fifth graders: Negative > Positive distance effect	14
		15	5	R	12y;8 m (11;9–13.5)	Seventh graders: Negative > Positive numbers	1
						Seventh graders: Positive distance effect	12
						Seventh graders: Negative distance effect	9
						Seventh graders: Positive > Negative distance effect	9
						Seventh graders: Negative > Positive distance effect	5
Kaufmann	2008	12	4	R	8.6 SD = 1.2	Nonsymbolic numerical processing	2
Kersey	2017	35	22	n/r	5.45 (3.6–6.99)	Conjunction:Adaptation and numerical deviant effect	5
Kucian	2011	15	7	n/r	10.6 SD 1.5	Non-Symbolic Numerical distance effect	14
Kucian	2011	16	n/r	n/r	9.5 SD 1.1	Order vs. control task	11
Libertus	2009	15	7	n/r	8.8 (8y 11 days–9y 1 mon)	Digits > (Letters and Faces)	3
Meintjes	2010	18	n/r	R	(8–12)	PJ > control task	17
Park	2014	21	12	R	5.55 (4.82–6.59)	All Number Tasks	16
						Numerical > Nonnumerical	7
						Symbol > Dots	9
						Close > Far	2
Vogel	2015	19	13	n/r	10.2 SD 2.55 (6–14)	Parametric modulation for number adaptation	5

Author	Year	Sample (N)	F	Hand	Age	Calculation Tasks	foci
Ashkenazi	2012	17	11	n/r	8.12 SD = 6.59mons	Complex > Simple Addition	23
Bertelletti	2015	39	22	n/r	11:4, SD = 1:6 (8:5–13:7)	Subtraction large vs small	6
Chang	2016	25	14	R	8.8 SD 0.7(7.7–10.7)	Subtraction − Control	14
Davis	2009	27	14	n/r	8.1 (7.1–9.4)	Single-digit exact calculation	5
						Double-digit exact calculation	13
						Single Digit Approximation	18
						Double Digit Approximation	8
Davis	2009	24	12	n/r	8.2 (8y1m–9y1m)	Exact calculation task	2
						Approximate calculation task.	7
De Smedt	2011	18	12	R	11.77 (10.08–12.92)	Small > large	11
						Large > small	8
						Addition > subtraction	1
						Subtraction > addition	15
Du	2013	19	n/r	n/r	10.62 SD 0.31 (10.12–11.26)	AP addition v EX addition (non trained group)	17
Iuculano	2015	15	8	n/r	(7.5–9.6)	Arithmetic problem solving before tutoring	3
Kawashima	2004	8	4	R	11.6 SD = 1.6 (9–14)	Addition	6
						Subtraction	6
						Multiplication	6
Krinzinger	2011	20	9	n/r	108 SD = 21 m (69–105 m)	Symbolic exact addition	12
						Non-symbolic exact addition	12
Meintjes	2010b	18	n/r	R	(8–12)	Exact addition > control task	25
Metcalf	2013	74	40	R	7.8 (7–9)	Complex > Control	6
Mondt	2011	24	13	R	9.6 SD = 0.9 (7.8–11.4)		
		16	n/r	R		3-Operand (Simple) Addition or Subtraction	2
		16	n/r	R		4-Operand (Complex) Addition or Subtraction	3
		8	n/r	R		3-Operand (Simple) Addition or Subtraction	5
		8	n/r	R		4-Operand (Complex) Addition or Subtraction	6
Peters	2016	22	10	5L	10.73 SD = 0.87 (9–12)	Subtraction: Digits − Fixation	6
						Subtraction: Words − Fixation	10
						Subtraction: Dots − Fixation	8
						Subtraction: Words − Digits	2
						Subtraction: Dots − Digits	10
						Subtraction: Dots − Words	10
						Subtraction: Digits − Dots	6
						Subtraction: Words − Dots	5
Prado	2014	34	21	n/r	11.54 (8.47–13.56)	Smaller subtraction	2
						Smaller subtraction > smaller multiplication	3
						Larger > smaller problems	2
						Larger > smaller multiplication,	4
						Smaller > larger (multiplication and subtraction combined).	4
Qin	2014	28	13	n/r	(7–11)	Addition > Control collapsing across Time-1 and Time-2	17
Rosenberg-Lee	2011	90	39	R	2nd graders 7.67 SD = 0.4; 3rd graders 8.67 SD = 0.4	Addition: Complex > Simple	5
						Addition: Simple > Complex	5

N = number of participants; F = females; Hand = handedness of participants; n/r = not reported; R = right handed; L = left handed Age = mean age, SD = standard deviation, and or age range in parenthesis.

### 1.2 Meta-analyses

Activation Likelihood Estimate (ALE) is a coordinate-based meta-analytic method (Turkeltaub et al., 2002, Eickhoff et al., 2009, Eickhoff et al., 2012). Foci from experiments (i.e., contrast coordinates) from selected articles are used to generate 3D maps indicating the likelihood of activation within a given voxel of a template brain (Eickhoff et al., 2009). Data are compared against a random spatial distribution (i.e., noise) to identify the likelihood of significant clusters. A whole-brain statistical map of ALE scores is generated that estimate the probability of the significance of a brain region being active during a given cognitive function.

ALE meta-analyses were computed using GingerALE 2.3.6 (http://brainmap.org/ale/). First, MNI coordinates were transformed into Talairach space using the best-fit MNI-to-Talairach transformation (Lancaster et al., 2007). Each meta-analysis (i.e., number and calculation tasks) contains foci from all possible relevant experiments as the analyses algorithm minimizes within-group effects and provides increase power (Turkeltaub et al., 2012, Eickhoff et al., 2017). We report brain areas that survived an uncorrected voxel-level threshold of p < 0.001 with a cluster-level correction at p < 0.05 for multiple comparisons (Eickhoff et al., 2012, Eickhoff et al., 2017). Contrast analyses are performed on images corrected for multiple comparisons with cluster level p = 0.05 and uncorrected p = 0.001, thus the threshold for contrast number tasks vs calculation tasks is set to p = 0.01 uncorrected, with 5000 permutations and minimum volume 50 mm^3^ (e.g., Sokolowski et al., 2017). For displaying results from ALE maps we employed AFNI (Cox, 1996).

For comparison we performed meta-analyses of fMRI data that examine number and calculation tasks with adults (methods and result presented in supplementary material).

## 2 Results

Mean age and/or age range of children in the meta-analyses for all experiments are shown on Table 1. The average age of children included in the meta-analyses was calculated to be 9.34 ± 2.18 years for number tasks and 9.58 ± 1.4 years for calculation tasks. Of the articles reporting gender, 44% and 52% of participants were female for number and calculation tasks, respectively. About 75% of the all articles reported handedness and tested right-handed participants with the exception of five left-handed participants in the sample for calculation tasks.

### 2.1 ALE maps

#### 2.1.1 Number tasks

**Fig. 2:**
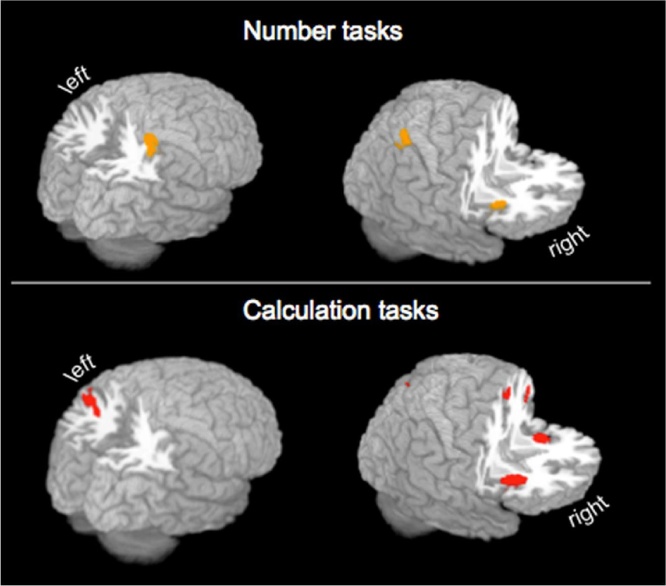
3D rendered ALE activation maps superimposed on an anatomical brain. All regions survived cluster level correction p = 0.05 for multiple comparison control at an uncorrected p = 0.001. All coordinates are listed in Table 2.

In children, number tasks are associated with significant ALE values in the inferior parietal lobule Brodmann area (BA 40) extending to parts of the inferior parietal sulcus, claustrum and insula (BA 13) in the right hemisphere (Table 2, Fig. 2).

**Table 2:** Concordant areas for processing number and calculation tasks in children.

	Volume mm^3^	ALE Value	x	y	z	Brain region
Children: Number tasks						
1	1736	0.019	38	−48	54	Right Inferior Parietal Lobule BA 40
		0.018	38	−46	42	Right Inferior Parietal Lobule BA 40
		0.012	34	−34	48	Right Postcentral Gyrus BA 3
2	1072	0.018	30	18	8	Right Claustrum
		0.015	36	16	0	Right Insula BA 13

Children: Calculation tasks						
1	4296	0.029	0	10	50	Left Superior Frontal Gyrus BA 6
		0.024	2	20	44	Right Medial Frontal Gyrus BA 8
		0.020	4	10	42	Right Cingulate Gyrus BA 32
2	3128	0.044	32	18	6	Right Insula BA 13
3	2848	0.030	−30	−62	38	Left Precuneus BA 19
		0.021	−42	−48	42	Left Inferior Parietal Lobule BA 40^a^
		0.018	−28	−72	42	Left Precuneus BA 19
4	1368	0.034	−30	16	6	Left Claustrum
5	1240	0.026	−46	4	36	Left Precentral Gyrus BA 6^b^
6	920	0.022	2	−70	48	Right Precuneus BA 7

Children: Number tasks > Calculation tasks						

No suprathreshold clusters						

Children: Calculation tasks > Number tasks						
1	984	3.540	2	8	40	Right Cingulate Gyrus BA 32
		3.156	−3	13	44.5	Left Medial Frontal Gyrus BA 6
		3.036	2	12	46	Right Medial Frontal Gyrus BA 6
2	528	2.948	3	−73	48	Right Precuneus BA 7
3	160	2.489	32	24	10	Right Insula BA 13
4	104	2.512	−30	12	6	Left Claustrum
		2.447	−32	16	6	Left Insula BA 13

Children: Calculation tasks AND Number tasks						
1	968	0.018	30	18	8	Right Claustrum
		0.015	36	16	0	Right Insula BA 13

Note: Single-study clusters survived a voxel-level threshold of uncorrected p < 0.001 with a cluster-level threshold for multiple comparisons at p < 0.05 (Eickhoff et al., 2017). Contrast threshold was set to p = 0.01, 5000 permutations, >50 mm^3^. Coordinates (x, y, z) are reported in Talairach convention; L, Left; R, Right; BA, Brodmann area; ALE, Activation likelihood estimate.

a Encompasses gray matter within angular gyrus BA 39.

b Encompasses gray matter in the inferior and middle frontal gyri (BA 9).

#### 2.1.2 Calculation tasks

When solving mathematical operations children activate a diverse set of areas (Table 2; Fig. 2). The largest cluster is centered on the left superior frontal gyrus (BA 6) and extends to the right medial frontal gyrus (BA 8) and cingulate gyrus (BA 32). The second largest cluster with the highest likelihood for being detected is found in the right insula (BA 13). Concordance in parietal areas is observed bilaterally; left precuneus (BA 19) and inferior parietal lobule (BA 40) including parts of the inferior parietal sulcus and angular gyrus BA 39, and right precuneus BA 7. The left claustrum has the second highest likelihood of being detected. A cluster centered on the left precentral gyrus (BA 6) encompasses also grey matter in the inferior and middle frontal gyri (BA 9).

#### 2.1.3 Contrast: number tasks vs calculation tasks

In children, compared to calculation tasks, number tasks show no suprathresholed clusters. Compared to number tasks, calculation tasks show increase concordance in the right cingulate gyrus (BA 32), bilateral medial frontal gyrus (BA 6), right precuneus (BA 7), bilateral insula and left claustrum, (Table 2). Areas that show concordance in conjunction between number and calculation tasks are the claustrum and insula (BA 13) in the right hemisphere (Table 2).

## 3 Discussion

Brain activity related to mathematical cognition in typically developing children was examined using coordinate-based ALE meta-analyses. Children activate a varied set of areas in established parietal and frontal regions when solving problems with numbers and operations. We highlight the role of insula and claustrum in mathematical operations because these regions have not been previously emphasized in this sort of cognition. To facilitate discussion, we organized findings by cortical regions, and for each we indicate characteristics of activity (e.g., its extent or magnitude) and the categories (i.e., number and calculation tasks), which elicit the activity. We build on previous models (Dehaene and Cohen, 1997, Arsalidou and Taylor, 2011) to offer a topographical model and a theoretical interpretation of mathematical cognition in children.

Number and calculation tasks elicit responses in the parietal lobes in children. This is a critical region linked to mathematical problem solving (Dehaene et al., 2003). Specifically, the first and most concordant cluster for number tasks is in the right inferior parietal lobule (BA 40), where complex (concrete or abstract) effort-demanding (non-automatized) objects are found (e.g., Pascual-Leone, 1995, Pascual-Leone and Johnson, 2005). The left inferior parietal lobule (BA 40) is activated during calculation tasks − sites for “active” complex objects that can receive transformations/operations (e.g., Pascual-Leone and Johnson, 2005). The critical role of the inferior parietal lobule in mathematical cognition has been discussed extensively in adults (Dehaene and Cohen, 2007, Dehaene et al., 2003, for reviews) and children (Cantlon et al., 2006, Davis et al., 2009b). The current findings are consistent with previous reports; and highlight a hemispheric dominance for number tasks in the right hemisphere, in contrast to calculation tasks driven by the left hemisphere.

When children solve mathematical operations, areas with the highest likelihood of being detected appear in the right insular cortex for calculation tasks. The left claustrum also shows one of the highest values of significant concordance for calculation tasks. The insula and claustrum are also concordant in a smaller cluster for number tasks. The insula is a structure that connects the temporal and frontal lobes deep within the lateral fissure; the claustrum borders the insula medially. Initially, the insula became known for its involvement in affective processes (for meta-analyses, see Gu et al., 2013, Duerden et al., 2013). Its role in the interaction of cognition, emotion and interoception has also been discussed (Uddin et al., 2014). Such interpretations of insular role are consistent with the hypothesis that, together with the anterior cingulate, insula may be involved in intrinsically motivated behaviours (Sridharan et al., 2008, Uddin and Menon, 2009, Pascual-Leone et al., 2015, Arsalidou and Pascual-Leone, 2016). The insula, together with subcordical structures such as the basal ganglia, have been implicated in studies that involve learning and training (Chein and Schneider, 2005). The role of the claustrum is often bundled with the functions of the insula; however, this is a distinct region anatomically (Mathur, 2014), and in terms of structural connectivity (Park et al., 2012). Recent reviews suggest the claustrum to function as a cross-modal integrator to create conscious percepts (Crick and Koch, 2005, Goll et al., 2015, for reviews). Thus, we propose that for children solving mathematical operations, high activation in the insula may express their *intrinsic motivation* in the task, with reference to learning and training. For the claustrum, we propose that it helps integrate motivated top-down and bottom-up processes. Importantly, neither the insula nor the claustrum have been implicated in models of mathematical cognition, although the current findings suggest that these areas are critical.

Another critical region for calculation tasks is the cingulate gyrus (BA 32), which extends medially from superior frontal (BA 6) and medial frontal (BA 8) gyri. The cingulate gyrus is also a key region for mental-attention and working memory, since it may be where affective intentions convert into cognitive goals (Arsalidou et al., 2013a, Pascual-Leone et al., 2015). Significant clusters of concordant activity in the dorsal cingulate gyrus (BA 32) are observed for calculation tasks in children. The cingulate gyri have been related to various higher order cognitive activities. Progressively more evidence highlights its role as a coordinator of activity in multiple attentional systems (Peterson et al., 1999), multimodal functions (Shackman et al., 2011), and as coordinating activity based on task complexity (Torta et al., 2013). We suggest that in children cingulate gyri integrate affective motives and available information to generate solutions involving specific cognitive goals.

Calculation tasks also elicit concordance in a cluster in the left precentral gyrus (BA 6) that extends to inferior and middle frontal gyri (BA 9). The middle frontal gyri, often referred to as dorsolateral prefrontal cortex, are associated with executive-driven mental attention and working memory (Arsalidou et al., 2013a, Christoff and Gabrieli, 2000, Christoff et al., 2009). Compared to inferior frontal cortices, which are associated with simpler cognitive actions, the dorsolateral prefrontal cortex implication in coordinated cognitive control of complex processes is important, and it was identified as such early on (Rypma et al., 1999, Christoff and Gabrieli, 2000, Christoff et al., 2009). Studies of mathematical cognition do associate activity in middle frontal gyri with procedural complexity and working memory processes (Delazer et al., 2003, Fehr et al., 2007, Kong et al., 2005, Simon et al., 2002, Zhou et al., 2007). This hypothesis is supported by adult data, which show that middle frontal gyri are implicated in calculation tasks but not in number tasks (Arsalidou and Taylor, 2011). We have replicated the latter finding with adult meta-analyses of number and calculation tasks (Table S2).

### 3.1 Topographical model of mental-arithmetic in children

**Fig. 3:**
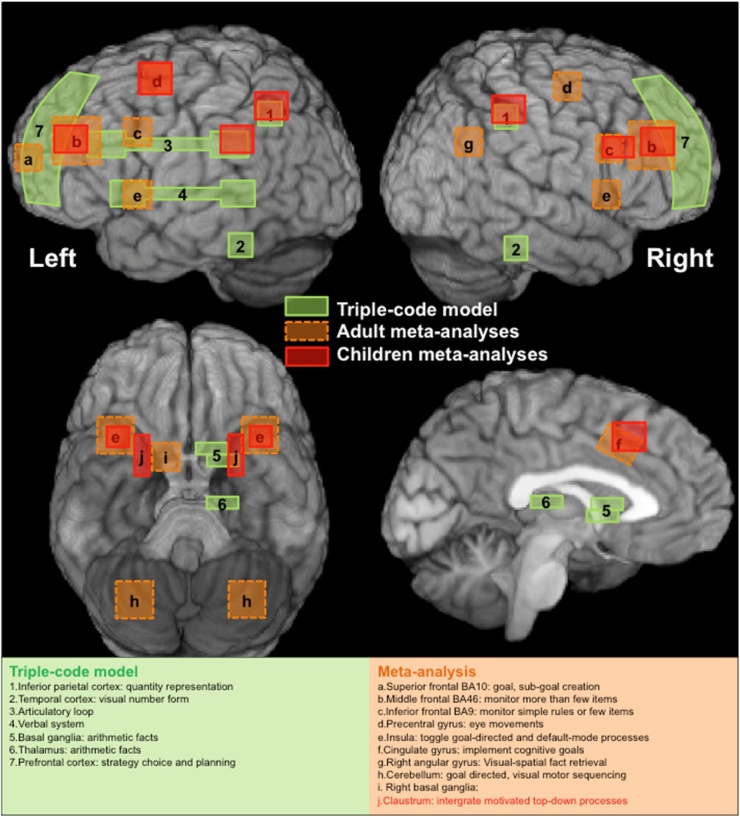
Mapping results on children meta-analyses (in red), on triple-code model (green), and adult meta-analyses (orange). We illustrate in green the schematized cortical locations of the triple-code model proposed by Dehaene and Cohen, 1995, Dehaene and Cohen, 1997: (1) Inferior parietal cortex: quantity representation, (2) Temporal cortex: visual-computational number symbols, (3) Articulatory loop, (4) Verbal system, (5) Basal ganglia: arithmetic facts, (6) Thalamus: arithmetic facts, and (7) Prefrontal cortex: strategy choice and planning. In orange are additional schematic locations of areas concordant among adult studies, as demonstrated by meta-analyses (Arsalidou and Taylor, 2011): (a) Superior frontal BA 10: formulates complex goals, sub-goal creation, (b) Middle frontal BA 46: in more or less misleading situations it monitors more than a few items, (c) Inferior frontal BA 9: monitor simple rules or a few items, (d) Precentral gyrus: eye movements, (e) Insula: interoceptive motivation of goal-directed and default-mode processes, (f) Cingulate gyrus: converts affective goals into cognitive goals to be implemented, (g) Right angular gyrus: visual-spatial fact retrieval (i.e., spatial-temporal schemes with non-verbalizable configural relations), and (h) Cerebellum: goal directed, visual motor sequencing. Sub-cortical regions specific to meta-analyses of number or calculation tasks were not depicted. Here we added the (i) right basal ganglia: coordination of top-down and bottom-up operative/motor processes. In red are schematic locations of areas concordant among children studies, as demonstrated by the current meta-analyses. (j) Claustrum: integration of motivated top-down and bottom-up processes.

There is a close agreement on brain locations implicated in children and adults during mathematical problem solving (Table S2–S3; Arsalidou and Taylor, 2011), and predictions by the triple-code model (Fig. 3). The only unaccounted brain region by either the triple-code model (Dehaene and Cohen, 1997) and the adult meta-analyses is the claustrum, which was concordant for children. A critical difference we observe is that children implicate the right insula (BA 13) more extensively than adults in calculation tasks, whereas adults implicate more prefrontal areas (BA 44, 46, 10; Table S3). We provide a theoretical framework that may explain these findings in the next section.

### 3.2 Developmental theory: the theory of constructive operators (TCO)

**Fig. 4:**
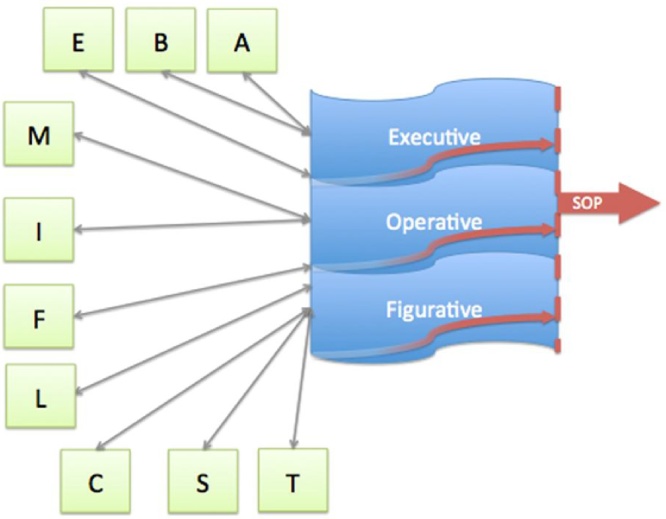
A simplified illustration of the Theory of Constructive Operators (TCO): Operators (in green; operator definitions are listed in Table 3), schemes (in blue), and the principle of schematic over-determination of performance − or SOP (in red).

The Theory of Constructive Operators (TCO) is a general, constructivist theory of development and learning (Pascual-Leone, 1995, Pascual-Leone, 1996, Pascual-Leone, 2014, Pascual-Leone and Baillargeon, 1994, Pascual-Leone and Johnson, 2005, Pascual-Leone and Johnson, 2011, Pascual-Leone and Johnson, 2017, Pascual-Leone et al., 2012). It provides a mathematical model for predicting developmental stages (Pascual-Leone, 1970, Pascual-Leone et al., 2010, Pascual-Leone et al., 2015, Arsalidou et al., 2010). The TCO is framed in terms of organismic *operators* (content-free brain utilities, i.e., regulations or general controls), *schemes* (contextual information carriers − cell assemblies or networks), and, finally, organizing *principles* (Pascual-Leone, 1969, Pascual-Leone, 1970, Pascual-Leone, 1995, Pascual-Leone and Johnson, 1991, Pascual-Leone and Johnson, 2005, Pascual-Leone and Johnson, 2011; Fig. 3). Operators are general-purpose and content free resources, which can apply on schemes in any domain. Table 3 provides a short description and basic brain location of operators in the TCO. Fig. 4 suggests how operators interact with schemes to elicit performance. Psychologically, schemes are self-propelling information-bearing units (cell assemblies or networks), which can be classified into three groups: executive, operative (action blue-prints), and figurative (object or feature representations). Thus, schemes carry information that can appear under three distinct categories: figurative, operative, and executive (Pascual-Pascual-Leone and Baillargeon, 1994; Fig. 3): (a) *figurative schemes* are perceptions and representations of concepts or objects (e.g., in the case of numbers: symbolic 3 or ★★★); (b) *operative schemes* correspond to actions and specific procedures, applicable to objects or concepts (e.g., in the case of math, the act of multiplying 3 × 3); and (c) *executive schemes,* a subdivision of operative schemes, that carry general contextualized procedures (e.g., in the case of math, to plan the order of steps for solving 3 + 3/(3 × 3)). The currently activated and dominant set of compatible executive schemes can function as an operator (which we call *E* for executive). *E* can regulate or control the current functioning of other operators (e.g., *M-* or *I-*operator; Table 3) as they apply to modify schemes in their here-and-now dynamism (e.g., Pascual-Leone and Johnson, 1991, Pascual-Leone and Johnson, 2005, Pascual-Leone and Johnson, 2011).

**Table 3:** Description of Operators and their Corresponding Brain Regions in their Likely Evolutionary Order (after Arsalidou, 2003; Pascual-Leone and Johnson, 2005).

Operator	Description	Brain Region
*A*	Set of **affective** processes that intervene in motivation and attentive arousal.	Limbic Lobes
*C*	Both the process of **content** learning and the schemes derived from associative content.	Primary & secondary association areas
*F*	The **field** operator together with the Schemes’ Overdetermination Principle (*SOP*, see below) act as the brain's *binding mechanism* bringing closure to mental representations in a neo-Gestaltist manner.	All Areas
*LC*	The process of automatized **logical-structural** learning derived from **content** learning through over-practice.	Right Hemisphere
*T*	**Temporarily** and effortlessly collates *sequences* of schemes, thus facilitating coordination of temporally-structured invariants that constitute distal objects.	Occipito-temporal
*S*	Facilitates emergence of **spatial** schemes by coordinating *relations of coexistence* among activated schemes effortlessly within the situation.	Occipito-parietal
B	Social ‘**Being**’ Schemes − related to personal, self/other referential schemes.	Default-mode areas
*I*	The attentional **interrupt**: It produces the central *active inhibition* of unwanted schemes, which were activated by the situation or the mind.	Prefrontal
*M*	Effortful **mental-**attentional *activation* of simple or complex (functional-structure) schemes.	Prefrontal
*LM*	**Logical-structural** learning caused by effortful use of **mental**-attentional capacity.	Left hemisphere tertiary areas
*E*	**Executive,** i.e., dominant *set of activated and compatible executive schemes* in the person’s repertoire that are useful for the task at hand.	Prefrontal

A principle of Schematic Overdetermination of Performance (*SOP −* Pascual-Leone and Johnson, 1991, Pascual-Leone and Johnson, 1999, Pascual-Leone and Johnson, 2005, Pascual-Leone and Johnson, 2011, expresses both the spreading of activation in the brain, and its *final common path* of neuronal resolution into a performance − which is a generalized version of Sherrington’s original idea (Sherrington, 1906, McFarland and Sibly, 1975). This *SOP* determines which set of compatible and dominant schemes eventually will apply to generate an outcome. Mutually incompatible schemes compete, and those more strongly activated eventually apply.

### 3.3 Integrating neuroimaging data and the TCO

Our findings have important theoretical and practical implications. Practically they provide coordinates in stereotaxic space that future studies may benefit from. Theoretically, they offer support for developmental theories that follow process-specific rather than content-specific approaches (Pascual-Leone, 1970, Pascual-Leone, 1995, Pascual-Leone and Johnson, 2011, Pascual-Leone and Johnson, 2017, Niaz and Logie, 1993). To clarify this issue we distinguish processes across four main dimensions of variation: (a) operative versus figurative processing, (b) complexity levels of processing, (c) left- versus right-hemisphere processing, and (d) motivation and self-control.

#### 3.3.1 Operative versus figurative

We suggest that operative processes and executive schemes are expressed in frontal and prefrontal areas, respectively, whereas representations and figurative/object schemes are expressed in parietal, temporal and occipital areas (Pascual-Leone, 1995, Pascual-Leone and Johnson, 2005). This is in agreement with early neurologists who viewed the brain as separating distinct, different modes of processing (e.g., Luria, 1970). It is important to explain why more operative processes and executive schemes are needed when solving calculations, expressed by additional brain activity in frontal and prefrontal areas. A clear distinction between the operative processes implicated in calculation tasks versus number tasks is found in adults (Table S2–S3), consistent with past meta-analyses (Arsalidou and Taylor, 2011). Operative processes are also important in children’s math tasks, as expressed by the involvement of prefrontal and sub-lobar regions, including insular cortex, during these tasks.

#### 3.3.2 Complexity levels in processing

Association areas may in some sense express level of *processing* required by a task. Consider that both *operative* (motor, efferent associative) and *figurative* (sensorial, afferent associative) schemes can be organized in context-sensitive *heterarchical* ways (i.e., functionally-flexible hierarchies). These heterarchies were first described by classic neuropsychologists (e.g., Luria, 1973, Eccles, 1980) as functionally nested sequences of *primary* areas (afferent or efferent − e.g., BA 4, and BA 1, 2, 3 and BA 17), *secondary* areas (coordination of local unimodal processes − e.g., BA 6, and BA 5, and BA 18), *tertiary* areas (coordination of *regional multimodal* areas − e.g., BA 8 and BA 7, BA 19), and finally *general multimodal* areas, also called *high tertiary or quaternary* (which are functionally generic, applicable across domains, and integrating the totality of regional multimodal processing − e.g., BA 9, BA 47, BA 46, BA 10, BA 39, BA 40). Distinguishing levels of processing complexity of a task is key for predicting and interpreting mathematical performance. In our data we find, for instance, that number tasks in children implicate secondary and tertiary association areas, whereas calculation tasks elicit activity in more tertiary areas.

#### 3.3.3 Left hemisphere versus right hemisphere processing

Traditionally semantic-pragmatic differences between left and right hemispheres, have been verbal-analytical versus visuospatial-global processing, respectively (Gazzaniga et al., 1962). However, this is no longer tenable as a main distinction between left versus right hemispheres − because we find that both hemispheres activate with both content types (i.e., verbal and visual-spatial). Within a constructivist-developmental viewpoint, we claim that propensities fostered by left hemisphere are best characterized as *analytical mental-attentional processing* (or novel, effortful working-memory) − often in *demanding* problem-solving situations; whereas right hemisphere fosters propensities of *overlearned or automatized processing* that engage in either very easy or very difficult tasks (Arsalidou et al., 2013b, Pascual-Leone, 1995, Pascual-Leone and Johnson, 2011). Table 4 shows circumstances that give rise to right or left hemisphere dominance by considering the novelty of task, mental-demand (*Md*) of the task and mental-attentional capacity (*Mc*) of the individual (Pascual-Leone and Baillargeon, 1994, Pascual-Leone and Johnson, 2011). A trade-off between *Md/Mc* can be used to predict hemispheric dominance such that when *Md << Mc* processing would favour the right hemisphere, when *Md *≤ *Mc* processing would favour the left hemisphere and when *Md >> Mc* processing would favour the right hemisphere. This distinction is novel with respect to the function of right hemisphere, which would be implicated in two different sorts of instances: (a) In more or less automatized (very easy) processes, or (b) when the needed mental-attentional resources are above and beyond what the individual has available (i.e., above his/her mental-attentional capacity; Pascual-Leone, 1989). In the latter case, when a task’s mental attentional demand is too high for the left hemisphere to cope with alone, the right hemisphere is mobilized in search of potentially useful overlearned or automatized schemes. For instance, in the current data (Table 2) we see that the largest cluster elicited in children when solving number tasks is in the right parietal cortex (since figurative schemes for number tasks are easier). In contrast, calculation tasks elicit activity mainly in the left parietal cortex (calculation involves harder − relational − figurative schemes to be coordinated using the child’s mental attentional capacity). Similarly, activity for the simple number tasks is observed in the right hemisphere, whereas calculation tasks implicate the left frontal cortex more extensively. It would be interesting for future studies to examine brain correlates of incorrect trials, to identify whether right hemisphere is a main contributor to attempts to solve trials that are too difficult for the children to solve.

**Table 4:** Model of Right-Left-Right hemispheric dominance.

Mc/Md trade off	Familiarity/Novelty	Hemisphere − strategy	Factors of transformation − Hemisphere
Md << Mc	High Familiarity	**Right** − associative heuristic to cope fast with easy task	with experience remains familiar −processing at Right
Md ≤ Mc	Novel	**Left** − mental attentional heuristics for problem solving	with experience becomes familiar, overlearned − transfer to Right
Md >> Mc	High novelty	**Right** − associative heuristic to cope with a too-difficulty task	with maturation Mc increases, and with experience task becomes less novel– transfer to Left

*Note:* Mc = mental-attentional capacity of the individual; Md = mental-attentional demand of the task.

#### 3.3.4 Motivation and self-control

Motivation is the process whereby *affective* tendencies are expressed in conscious or unconscious affective goals (motives); which are then converted into conscious or unconscious cognitive goals (Pascual-Leone and Johnson, 2004, Arsalidou and Pascual-Leone, 2016, Arsalidou et al., 2017). *Affective goals* are dispositions to pursue desirable vital/life outcomes or consequences, whereas *cognitive goals* are explicit intentions to do what is believed congruent with one’s affective goals. Motivation crosses borders between affective and cognitive goals, and is critical for successfully doing difficult mental tasks. We propose that these affectively-driven *endogenous* cognitive goals rely critically on the insular cortex.

The insula is a structure that connects temporal and frontal lobes deep within the lateral (Sylvian) sulcus; it is active in many diverse task situations, including cognition, interoception, perception and emotion (Cauda et al., 2012, Uddin et al., 2014, for meta analyses). It is also implicated in cognitive processes such as inhibitory control (Cai et al., 2014), and speech and language processing (Oh et al., 2014). In a study examining math anxiety, data showed that anticipating an upcoming math-task elicited increased activity in the posterior insular cortex (Lyons and Beilock, 2012). Together with sub-cortical structures like the basal ganglia, the insula is involved in learning and training tasks (Chein and Schneider, 2005, Thomas et al., 2004, Ferreira et al., 2015). It has been shown to participate in the affective − aversive − feeling of difficult effort (Damasio, 2010, Naqvi and Bechara, 2010, Yu et al., 2010). The insula integrates interoceptive affect feedback and is related to the orbitofrontal cortex − involved in exteroceptive feedback (Holroyd and Yeung, 2011). Regarding interoceptive feedback, Damasio writes: “… relative to the visceral and internal milieu, the insula is the equivalent of the primary visual or auditory cortices” (Damasio 2010, p.125). The insula is proposed to have a generic role in problem solving (Arsalidou and Taylor, 2011), and mathematics may serve as a domain for targeted testing of the understanding of interceptive feeling of effort when comparing children and adults.

Self-control is related to motivation, in the sense that a motivated action requires self-control to be carried out, particularly in complex situations. When task problem-solving appears complicated and misleading, self-consciousness may become particularly necessary (e.g., Damasio, 2010, Pascual-Leone, 2000). The operative aspect of this self (i.e., the functional organization that William James would call I-self) is possibly in medial prefrontal cortex and anterior cingulate cortex; this is the operative aspect of self-control found in the *medial frontal cortex* (Arsalidou et al., 2013b, Rottschy et al., 2012, Pascual-Leone et al., 2015). The figurative/representational self-processes (the me-self of William James) would be located (see Damasio, 2010), in the *posteromedial cortex*, i.e., the combination of posterior cingulate cortex, retrosplenial cortex, and precuneus.

The active coordination between motivation and self-control would elicit activation in the anterior cingulate and insular cortices. Both anterior cingulate and insular cortices are prevalent clusters in children’s mathematical problem-solving. In adults, the cingulate and insula cortices activate bilaterally in number and calculation tasks (Table S2). Importantly, these activations in adults are listed further down in the ALE-obtained coordinate list (Table S2 cluster 3 and 4 for number tasks and cluster 5 and 7 for calculation tasks), possibly because adults’ prior practice has already transformed into cognitive goals and cognitive circuits/networks the original affective goals (motives) *and* interoceptive feelings of effort, within the math domain. In contrast, the cingulate gyrus and the insula occupy in children respectively the first and second larger clusters of activity for calculation tasks; moreover the insula has the highest likelihood of being detected during calculations in children. In our view this finding is important, suggesting that intrinsic (interoceptive) motivation and self-control in children are critical to perform difficult calculations − but much less so during number tasks, which are more automatized.

### 3.4 Limitations and considerations for future studies

The current meta-analyses focus on functional correlates of brain regions in children relating to number and calculation tasks. We report data representing concordance across these types of task. There are limitations associated with the meta-analysis method, and with choices we had to make, due to the methodology in the original studies. Shortcomings of the ALE method have been previously discussed (e.g., Di Martino et al., 2009, Arsalidou et al., 2013b). However, ALE meta-analysis remains superior to traditional review approaches, because it permits investigating over-arching patterns of activity across studies. An advantage is the automated computational steps that permit quantification of locations of common activation across studies. In terms of article selection, we chose a conservative approach; we eliminated many articles that appeared to use same participants (see method section), and articles that used children in large age ranges. Eliminating these studies may have lowered strength of concordance detected, but the conclusions drawn from such conservative approach are likely to be more representative of brain responses in school age children, which was our main concern. Further, because of a lack of studies, we have not specifically examined brain responses to mathematical operations separately (i.e., only addition, only multiplication). Methodological choices in some studies lacked developmental theoretical grounding; for instance, since we know from behavioural research that performance on mathematical abilities improves with age, studies should specify a theoretical reason for averaging data over large age ranges. Similar behavioural performance on a task does not imply that the same set of brain regions support the particular performance across age groups (Arsalidou and Pascual-Leone, 2016). For future studies, in addition to demographics such as gender and age range of participants (missing in some articles), it is critical to report within-group contrast coordinates for healthy children − even when this is not the main focus of the paper. Such practices would be beneficial for future meta-analyses on the topic.

## 4 Conclusion

These meta-analyses investigate brain activity in children that underlies processing of number and calculation tasks. These are the first meta-analyses in children younger than 14 years distinguishing between number and calculation tasks. Based on these results we sketched a neuropsychological developmental model of mathematical cognition in stereotaxic-space. We find that mathematical performance in children emerges from known core-regions associated with number processing, such as parietal and frontal areas; but it also emerges from regions not previously recognized in a mental-arithmetic network, such as the insula, the claustrum, and the cingulate gyrus. The insula, in particular, may play a critical role in children’s mathematical calculation, because children need strong intrinsic motivation and affective goals to cause their effort in attention and complex processing. Future behavioural and neuroimaging work on children’s mathematical cognition should benefit from a refined topographical atlas of mathematical processes in healthy children.

## Conflict of interest

None.
