# Supplementary material for: Brain areas associated with numbers and calculations in children: Meta-analyses of fMRI studies
Source: Dev Cogn Neurosci. 2017 Aug 8;30:239–50. doi: 10.1016/j.dcn.2017.08.002 (PMC6969084; doi:10.1016/j.dcn.2017.08.002)
Supplement: Supplementary file 1 [file mmc1.pdf]

SUPPLEMENTARY MATERIAL FOR:

Brain areas associated with numbers and calculations in children: Meta-analyses of fMRI studies

Marie Arsalidou[1, 2, *], Matthew Pawliw-Levac[1], Mahsa Sadeghi[1], Juan Pascual-Leone[1]

[1] Department of Psychology, Faculty of Health, York University, Toronto, Canada

[2] Department of Psychology, National Research University Higher School of Economics, Moscow, Russian Federation

[*]Corresponding Author: Marie Arsalidou

E-mail: marie.arsalidou@gmail.com

Phone: +1 786 505 9779

Behavioural Science Building

York University

4700 Keele Street,

Toronto, Ontario, Canada

M3J 1P3

ADULT META ANALYSES

Methods

The literature was searched using web-of-science (http://www.isiknowledge.com) in June 2017 with keywords (fMRI and arithmetic, calculations, math, and mathematics). The search yielded 1191 articles, which were then checked for duplicates, sorted and subjected to a series of selection criteria. In order to be included in the analyses, articles had to (a) be written in English, (b) use fMRI and involve tasks of mathematics or numbers, (c) include healthy participants, (d) report whole-brain, within-group results using random-effects analysis, and (e) report stereotaxic coordinates in Talairach or Montreal Neurological Institute (MNI) space. Figure 1 indicates the steps taken and number of studies included in the meta-analyses. Only those articles with adult participants (age range 18-45) were included. Articles identified as reporting results from participants with similar characteristics (i.e. same number of participants, mean age, and gender distribution), namely those by the same authors within close chronological order, were also excluded. Data from 115 articles are included in the meta-analyses.

Experimental paradigms were categorized as either number tasks or calculation tasks. *Number tasks* were those that involved numerosity and numerical processing without formal operations (i.e. numerical Stroop, distance effects, quantitative comparisons), while *calculation tasks* involved formal operations (i.e. addition, subtraction, or multiplication). Each meta-analysis contains foci from all possible relevant experiments (i.e., contrasts) as the algorithm that minimizes within-group effects (Turkeltaub et al., 2012; Eickhoff et al., 2017); experiments were organized by subject group. Three examiners (MS; MPL; MA) undertook this selection process separately, and then came to final agreement. Table S1 shows article information, participant demographics and contrast selection for each category.

Meta-analyses

Activation likelihood Estimate (ALE) meta-analyses were computed using GingerALE 2.3.6 (http://brainmap.org/ale/; Eickhoff et al., 2017). First, all contrast coordinates (i.e. foci) from original articles were transformed into a Talairach common space using the best-fit MNI-to-Talairach transformation (Lancaster et al., 2007). Significance was determined with uncorrected p-value < 0.001, with cluster-level correction of p = 0.05 for

multiple comparisons (Eickhoff et al., 2012; 2017). Contrast analyses are performed on images corrected for multiple comparison control with cluster level p = 0.05 and uncorrected p = 0.001, thus the threshold for contrasts Number tasks: Adults vs Children and Calculation tasks: Adults vs Children is set to p = 0.01 uncorrected, with 5000 permutations and minimum volume 50 mm^3 (e.g., Sokolowski et al., 2017).

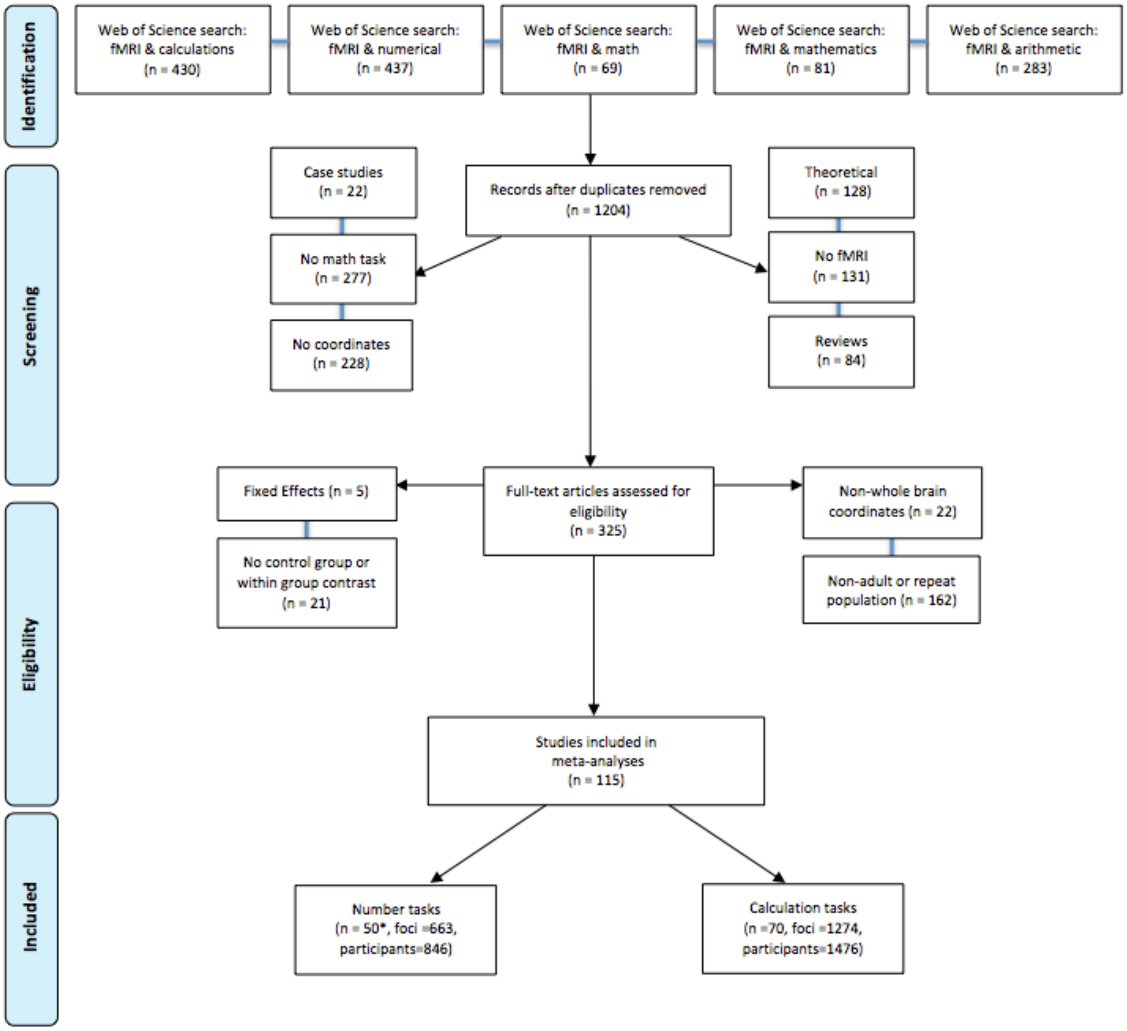

Figure S1. PRISMA flowchart for identification and eligibility of articles for adult meta-analyses (template by Moher et al., 2009). n = number of papers. *One paper (LeClech et al., 2000) reported data on two separate groups of participants, thus two experiments were included for this article.

Table S1. Descriptive information of articles and contrasts used in the adult meta-analyses.

Author	Year	N	F	Hand	Age	Calculation tasks	Foci
Anderson et al.	2014	39	NR	R	NR	Linear Trend Mastery	6
Andin et al.	2015	17	12	R	22-37	random multiplication > visual control	8
						subtraction > visual control	4
						Multiplication > phonology	3
						subtraction > phonology	2
Andres et al.	2011	10	0	R	21	Multiply and Subtract	8
Andres et al.	2012	18	0	R	21	Multiplication > Subtraction	2
Ansari et al.	2011	24	0	R	26.83	arithmetic problems-incorrect > correct	8
Audoin et al.	2005	10	NR	R	26.6	PASAT Task > Rest	47
Benn et al.	2014	20	10	R	18.9	Harbormaster Game Condition 4 > 1	16
Bulthe et al.	2014	16	12	15 R	21-28	Subtraction Localizer	19
Chochon et al.	1999	8	4	R	22.3	Multiplication > Control	12
						Subtraction vs. Control	14
						Multiplication vs. Digit Naming	4
						Subtraction vs. Digit Naming	11
Dehaene et al.	1999	7	3	R	22-28	Exact > Approximate Addition	7
Delazer et al.	2003	13	6	R	30.5	Untrained Multiplication > Number Matching	13
						untrained > trained multiplication	13
Delazer et al.	2004	13	NR	R	NR	Untrained Multiplication > Trained	5
DePisapia et al.	2007	20	12	R	20.3	Mental Arithmetic	9
DeVisscher et al.	2015	20	10	R	23-34	Large > Small Problems	7
Fehr et al.	2007	11	6	11 R	26.8	Conjunction of Operations	7
						Addition- complex>simple	17
						Subtraction - complex>simple	19
						Multiplication - complex>simple	9
						Division - complex>simple	15
Feng et al.	2014	20	9	R	21.65	2-relational > 0-relational	9
						1-relational > 0-relational	8
Grabner et al.	2012	29	14	R	23.45	Untrained Multiplication & Subtraction > Trained	4
Grabner et al.	2009	28	0	R	26.86	Untrained Multiplication > Trained	15
Grabner et al.	2007	25	0	NR	25.6	Multi Digit > Single Digit	12
Grabner et al.	2013	30	15	R	22.7	Small > Large Equations (Correct)	3
Gullick et al.	2014	24	12	R	22.6	Addition > Subtraction	12
Hanakawa et al.	2003	16	8	15 R	22-34	Mental Operation Numerical Task > VR Task	9
Harada et al.	2013	24	24	R	20.4	Exact Addition > Control	12
Hsu et al.	2016	20	9	R	22.8	~Value ∩ Math ∩ ~Emotion	7
Hugdahl et al.	2007	12	12	R	31	Continuous Addition with Controls	11
Ischebeck et al.	2009	17	7	R	25	Untrained Multiplication > Trained	17

Ischebeck et al.	2006	12	8	NR	26.8	Multiplication Untrained	13
						Subtraction untrained	21
Ischebeck et al.	2007	18	9	R	27.8	1st Third: Novel > Repeated Multiplication	2
Jost et al.	2009	17	10	R	24.5	Small Multiplication > Storage	16
Kanjlia et al.	2016	19	9	NR	46	Math > Sentences	15
Kawashima et al.	2004	16	8	R	44.1	Multiplication	10
						Addition	10
						Subtraction	8
Keller & Menon	2009	49	25	R	23.99	Calculation > Identification	9
Klein et al.	2010	17	0	R	28	Conjunction increasing decade sum, unit sum	15
						Main effects of problem size and carry and their interaction	15
Kong et al.	2005	16	9	R	28	Subtraction with Borrowing	10
						Subtraction without borrowing	11
						Addition without carrying	5
						Addition with carrying	14
Krueger et al.	2008	18	5	R	25.3	Integration Problem > Font	12
Kuo et al.	2008	12	6	R	21-29	Dual operation > Baseline	26
						Single-addition > Baseline	13
						Single-subtraction > Baseline	17
						Dual-addition > Baseline	15
						Dual-subtraction > Baseline	21
Landro et al.	2001	12	NR	NR	20-45	Addition > Off Block	5
						add unitl 2 digits = 10 > respond to #7	4
Lee et al.	2000	11	5	R	25-35	Multiplication > Subtraction	6
Liang et al.	2016	15	8	R	22.1	Calculation > Reasoning	7
Liu et al.	2017	30	15	R	22	Computation > Language	32
						Computation	17
Menon et al.	2000	16	8	R	20.28	3 second rate, 3-operand	16
						3 second rate, 2-operand	6
Molko et al.	2003	14	NR	NR	24.5	Calculation > Rest	11
Newman et al.	2011	15	9	R	22	Number Hard problems	10
						Number easy problems	9
Pinel et al.	2013	64	0	R	22.79	Calculation network	22
Pletzer et al.	2016	74	34	R	25.4	Subtraction - all	4
						Multiplication - all	3
Prabhakaran et al.	2001	7	3	R	26	2 operation > none	103
Prado et al.	2013	53	30	NR	25.2	Large Multiplication > Small	5
Rickard et al.	2000	8	5	R	24	Multiplication Verification	8
Salimpoor et al.	2010	41	21	NR	22.6	Novel Mathematical Calculation > Repeat	10
Sammer et al.	2007	20	10	R	25.4	Mental Arithmetic > Reference	8
Simon et al.	2002	10	7	R	25	Calculation > Control	23
Soylu et al.	2016	13	6	R	24.67	Main effect of addition difficulty	4

Stanescu-Cosson et al.	2000	7	3	R	22-26	All Calculation > Letter Matching	16
						exact > approximation	12
Tallian et al.	2015	18	NR	R	19-30	Multiplication Rounding Choice > No-Choice	8
Tan et al.	2007	22	9	R	NR	Numerical Computation CJ > J	38
Tenison et al.	2014	20	10	R	21.8	Trained > Untrained problems	6
Tschentscher & Hauk	2014	26	13	R	NR	Arithmetic Fact Retrieval > Procedural Strategies	6
Ulrich et al.	2016	23	0	R	24	Addition: Flow > Boredom & Overload	29
Vassena et al.	2014	22	14	NR	18-24	High Effort > Low Effort	11
Venkatraman et al.	2006	20	7	R	20-25	Base-7 Addition Task	18
Wang et al.	2007	19	12	R	36	Calculation (native Chinese speakers)	8
Wang et al.	2005	18	9	NR	22-36	Continuous Subtraction	7
						Results of repeated recitation of the multiplication tables	5
Wessa et al.	2013	30	17	26 R	21.8	Arithmetic > Number Detection	14
Wood et al.	2008	20	0	R	24.2	Multiplicative > Non-Multiplicative	2
Wu et al.	2009	18	11	NR	22.3	Calculation > Identification	3
Yang et al.	2013	17	7	R	25.76	Addition and Subtraction	18
YiRong et al.	2011	32	16	R	24.25	Conjunction of Addition & Subtraction	7
Zago et al.	2008	14	8	R	20-27	Numbers Manipulation (Addition) > Maintenance	17
Zarnhofer et al.	2012	42	21	R	23	Multiplication > Subtraction	8
Zhou et al.	2007	20	10	R	22.7	Multiplication Large	16
						Addition Large	15
						Addition Small	15
						Multiplication Small	16
**Author**	**Year**	**N**	**F**	**Hand**	**Age**	**Number Tasks**	**Foci**
Anderson et al.	2015	15	8	R	24.8	Parity > Reading	4
Ansari et al.	2005	12	NR	NR	19.8	Small > Large Distance	11
Ansari et al.	2007	13	NR	R	21.5	Small > Large Symbolic symbolic	8
						small > large nonsymbolic	3
Ansari et al.	2006	14	8	R	21.4	Small > Large Distance	9
Cantlon et al.	2006	12	11	NR	25	Number > Shape	2
Cavdoroglu et al.	2015	14	10	R	26.3	Visual Numerocity > Rest	8
						Visual ResponseVN>R	17
Chen et al.	2007	20	10	R	22.7	Unmatched > Matched Numbers	8
Chiao	2009	12	6	R	20.7	Number Comparison	5
						Number distance	8
Chochon et al.	1999	8	4	R	22.3	Digit Comparison > Control	13
						Digit Naming vs. Control	2
Cohen-Kadosh et al.	2005	15	7	NR	27.8	Numerical > Size	7
						Numerical vs. luminance 8	8
						Numerical distance 3	3
Cohen-Kadosh et	2007	14	8	R	25.6	Size Congruity Effect	17

al.							
Critchley et al.	2005	15	NR	R	23	Incongruent > Congruent Stroop	5
Daniels et al.	2003	8	4	R	25.4	Random Number Generation 1Hz	19
						Random Number Generation 2Hz	7
Demeyere et al.	2014	12	9	11 R	26	Small > Large	9
Dormal et al.	2010	15	0	R	21	Simultaneous Numerosity	6
						Sequential Numerosity	6
Eger et al.	2003	9	5	R	27.9	Numbers > Letters and Colours	4
						Numbers > Colours	3
Emerson et al.	2015	20	NR	NR	NR	Number-Selective Activation	5
Franklin et al.	2009	17	10	R	21.8	Magnitude Near > Far	3
Gobel et al.	2004	12	6	6 R	26.7	Number Comparison > Rest	37
Gullick et al.	2011	17	8	R	21.4	Close Distance > Far	18
He et al.	2014	20	8	R	21	Large > Small Problems	6
Holloway et al.	2010	19	10	R	23.5	Symbolic > Non-Symbolic	6
Hung et al.	2015	40	17	R	19-30	Main Effect of Number Words	42
Kansaku et al.	2007	13	7	R	20-42	Large Number Counting	7
						Small Number counting	7
Kaufman et al.	2005	14	5	R	31.1	Numerical Comparison > Null Events	14
						Numerical comparison > physical comparison	5
Kaufman et al.	2008	12	6	R	33.2	Non-symbolic Numeral > Baseline	2
Landro et al.	2001	12	NR	NR	20-45	Respond to 7 > Off Block	6
Leibovich et al.	2016	19	12	R	22.75	Task effect: numerical > nonnumerical	2
LeClech et al.	2000	5	0	NR	37	Exp: 1 Numerals > Body Parts	4
LeClech et al.	2000	6	0	NR	27	Exp: 2 Numerals > Body Parts	3
Leibovich et al.	2015	40	16	NR	24.4	Numerosity Task > Continuous Task	4
Liu et al.	2006	12	7	R	18-45	Numerical Processing	2
Ogata et al.	2011	13	4	R	21.7	Long Choice > Short Distance	8
						Long Choice > Long Distance	4
						Short Choice > Long Distance	8
						Short Choice > Short Distance	9
Piazza et al.	2006	10	3	R	23-31	Counting > Matching	12
Piazza et al.	2004	12	NR	R	23	Deviations in Number	7
Piazza et al.	2007	14	NR	R	NR	Far > Close Distance	21
Pinel et al.	1999	11	2	R	25.7	Number Comparison Task	47
Pinel et al.	2001	26	10	R	25	Distance Effect	7
Pinel et al.	2004	15	10	R	23.7	Number Comparison > Size Comparison	5
Prado et al.	2011	26	16	R	19-30	Numerosity Comparison (Localizer Task)	7
Prado et al.	2013	53	30	NR	25.2	Hard Comparison > Easy	4
Robertson et al.	2015	16	8	R	19-27	Incongruent > Congruent	42
Skagerlund et al.	2016	24	14	R	24.33	Number > Control	6
Leroux, Speiss et al.	2009	9	0	R	23.2	Numerical Judgement > Colour Detection Reference	10
Tan et al.	2007	24	9	R	NR	Numerical Size Judgement > Motor Task	8
Tang et al.	2006	18	7	R	25	Numerical Distance > Physical	10

						Distance	
Tschentscher et al.	2012	29	15	R	25.1	Numbers > Baseline	6
Vogel et al.	2013	14	7	R	24	Number Line Estimation > Word Control Task	10
						number specific >control	5
Wood et al.	2006	14	0	R	27	Decade Distance	11
Wood et al.	2008	20	0	R	24.2	Large Bisection Range > Small Range	18
Zhou et al.	2006	12	6	R	21.4	Numerical Sequence Activation (Forward) > Fixation	15
						Number > Alphabet	13

N = number of participants; F = females; Hand = handedness of participants; NR = not reported; R = right handed; L = left handed Age = mean age, SD=standard deviation, and or age range in parenthesis.

Results

Number tasks

Number tasks in adults are associated with significant ALE scores mainly in the parietal cortex bilaterally: superior and inferior parietal lobules (BA 7 and BA 40; Table S2). Other areas include the cingulate gyri (BA 32, BA 24), inferior frontal gyrus (BA 9) and insula.

Calculation tasks

Calculation tasks in adults elicit concordant activity in parietal and prefrontal regions bilaterally: precuneus (BA 7), inferior and superior parietal lobules (BA 7 and BA 40) and inferior and middle frontal gyri (BA 9, BA 46, BA 10; Table S3). Other areas include the superior frontal gyrus BA 6, the cingulate gyrus and the insula bilaterally.

Contrast: Adults vs Children

For number tasks, the conjunction between adults and children show significantly increased ALE values in the inferior parietal lobule (BA 40) and insula (BA 13) in the right hemisphere (Table S3). For number tasks, adults more significant clusters in parietal areas and (BA 7 and 40) and the cingulate gyrus (BA 32); whereas no suprathreshold clusters were observed in the contrast children > adults.

For calculation tasks, a conjunction between children and adults elicits concordance in

parietal and frontal areas mainly in the left hemisphere (Table S3). Adults show greater concordance mainly in prefrontal regions such as the inferior and middle frontal gyri in BA 44 and BA 46, whereas children show greater concordance in areas such as the insula, precuneus, cingulate gyrus and claustrum (Table S3).

Table S2. Concordant areas for processing number, calculation tasks in adults.

Adults: Number tasks						
#	Volume mm^3	ALE Value	x	y	z	Brain area
1	12776	0.051	38	-48	48	Right Inferior Parietal Lobule BA 40
		0.048	30	-52	44	Right Superior Parietal Lobule BA 7
		0.045	24	-64	42	Right Superior Parietal Lobule BA 7
		0.043	26	-66	36	Right Precuneus BA 7
		0.024	10	-72	52	Right Precuneus BA 7
2	11416	0.052	-42	-40	42	Left Inferior Parietal Lobule BA 40
		0.042	-26	-64	42	Left Superior Parietal Lobule BA 7
		0.034	-26	-54	42	Left Superior Parietal Lobule BA 7
3	6472	0.043	2	12	50	Right Superior Frontal Gyrus BA 6
		0.034	6	22	38	Right Cingulate Gyrus BA 32
		0.031	-8	6	46	Left Cingulate Gyrus BA 24
4	2672	0.029	34	16	10	Right Insula BA 13
		0.026	30	20	4	Right Claustrum
		0.024	36	18	-2	Right Extra-Nuclear BA 47
5	1856	0.033	48	8	26	Right Inferior Frontal Gyrus BA 9
6	1376	0.031	-46	2	28	Left Precentral Gyrus BA 6
7	904	0.026	-34	16	-2	Left Insula BA 13
		0.018	-32	16	8	Left Insula BA 13

Calculation tasks: Adults						
#	Volume mm^3	ALE Value	x	y	z	Brain area
1	14536	0.088	-26	-66	36	Left Precuneus BA 7
		0.068	-28	-58	46	Left Superior Parietal Lobule BA 7
		0.064	-40	-46	40	Left Inferior Parietal Lobule BA 40
		0.063	-36	-48	40	Left Inferior Parietal Lobule BA 40
		0.032	-12	-70	48	Left Precuneus BA 7
2	10664	0.089	30	-54	40	Right Superior Parietal Lobule BA 7
		0.063	38	-44	40	Right Inferior Parietal Lobule BA 40
		0.033	14	-70	44	Right Precuneus BA 7
3	8768	0.096	-46	6	30	Left Inferior Frontal Gyrus BA 9
		0.060	-44	30	26	Left Middle Frontal Gyrus BA 9
4	6944	0.068	44	6	30	Right Inferior Frontal Gyrus BA 9
		0.047	42	32	24	Right Middle Frontal Gyrus BA 46
		0.027	42	44	24	Right Middle Frontal Gyrus BA 10
5	6368	0.093	-4	10	50	Left Superior Frontal Gyrus BA 6
		0.040	6	22	36	Right Cingulate Gyrus BA 32
		0.028	-4	-6	60	Left Medial Frontal Gyrus BA 6
6	3472	0.069	-28	-2	56	Left Sub-Gyral BA 6
7	3432	0.094	30	20	6	Right Insula BA 13
8	3232	0.078	-30	22	4	Left Insula BA 13
9	2792	0.036	-48	-54	-14	Left Fusiform Gyrus BA 37
		0.032	-44	-64	-12	Left Fusiform Gyrus BA 37

		0.028	-40	-76	-8	Left Middle Occipital Gyrus BA 18
10	2208	0.039	32	-84	-6	Right Inferior Occipital Gyrus BA 18
		0.032	18	-90	-4	Right Lingual Gyrus BA 17
11	2016	0.053	26	-2	56	Right Sub-Gyral BA 6
12	1168	0.030	-24	-86	-4	Left Middle Occipital Gyrus BA 18
		0.028	-18	-92	-6	Left Inferior Occipital Gyrus BA 17
13	920	0.032	-30	-58	-34	Left Cerebellar Tonsil

Note: All clusters survived a cluster-level threshold for multiple comparisons at $p < 0.05$ and a voxel-level threshold of uncorrected $p < 0.001$ (Eickhoff et al., 2012, 2017). Coordinates (x, y, z) are reported in Talairach convention; BA, Brodmann area; ALE, Activation likelihood estimate.

Table S3. Between groups contrasts and conjunction analyses for number and calculation tasks.

Number tasks: Adults_AND_Children

#	Volume mm^3	ALE Value	x	y	z	Brain area
1	1744	0.019	38	-48	54	Right Inferior Parietal Lobule BA 40
		0.018	38	-46	44	Right Inferior Parietal Lobule BA 40
		0.016	40	-52	48	Right Inferior Parietal Lobule BA 40
2	1120	0.021	30	18	8	Right Claustrum
		0.015	36	16	0	Right Insula BA 13

Number tasks: Adults > Children

#	Volume mm^3	ALE Value	x	y	z	Brain area
1	1984	3.540	26.1	-54	41.4	Right Precuneus BA 7
		3.353	26.8	-55.3	35.2	Right Precuneus white matter
2	344	2.911	-6	8	42	Left Cingulate Gyrus BA 32
		2.820	-10	8	42	Left Cingulate Gyrus BA 32
3	96	2.457	-34	-34	40	Left Inferior Parietal Lobule BA 40
		2.400	-32	-34	44	Left Inferior Parietal Lobule BA 40

Number tasks: Children > Adults

No suprathreshold clusters

Calculation tasks: Adults_AND_Children

#	Volume mm^3	ALE Value	x	y	z	Brain area
1	2776	0.030	-30	-62	38	Left Precuneus BA 19
		0.021	-42	-48	42	Left Inferior Parietal Lobule BA 40
		0.018	-28	-72	42	Left Precuneus BA 19
2	2776	0.029	0	10	50	Left Superior Frontal Gyrus BA 6

		0.023	4	20	42	Right Cingulate Gyrus BA 32
3	2264	0.044	32	18	6	Right Insula BA 13
4	1064	0.026	-46	4	36	Left Precentral Gyrus BA 6
5	992	0.034	-30	16	6	Left Claustrum

Calculation tasks: Adults > Children

#	Volume mm^3	ALE Value	x	y	z	Brain area
1	632	2.878	52	12	22	Right Inferior Frontal Gyrus BA 44
2	304	2.878	-50	6	20	Left Inferior Frontal Gyrus BA 44
3	272	2.489	-42	38	24	Left Middle Frontal Gyrus BA 46
		2.478	-46	36	26	Left Middle Frontal Gyrus BA 46
		2.428	-48	33	22	Left Middle Frontal Gyrus BA 46
4	240	2.636	-24	-60	50	Left Precuneus BA 7
		2.605	-24	-56	52	Left Precuneus BA 7
5	80	2.605	24	-58	44	Right Superior Parietal Lobule BA 7

Calculation tasks: Children > Adults

#	Volume mm^3	ALE Value	x	y	z	Brain area
1	1040	3.540	34	24	14	Right Insula BA 13
		3.353	36	20	12	Right Insula BA 13
		3.239	38	15	9	Right Insula BA 13
2	832	3.540	1.1	-66.2	46.7	Left Precuneus BA 7
		3.353	1.4	-71.1	48.9	Left Precuneus BA 7
3	232	2.878	7.3	12	41.3	Right Cingulate Gyrus BA 32
4	144	2.848	-28	14	6	Left Claustrum
5	80	2.669	2	24	44	Right Medial Frontal Gyrus BA 8
6	72	2.687	32	-58	-30	Right CerebellumTuber

Note: Single-study images used for the contrast survived a voxel-level threshold of uncorrected $p < 0.001$ with a cluster-level threshold for multiple comparisons at $p < 0.05$ (Eickhoff et al., 2017), thus the threshold for constasts was set to $p = 0.01$, 5000 permutation, > 50 mm^3. Coordinates (x, y, z) are reported in Talairach convention; L, Left; R, Right; BA, Brodmann area; ALE, Activation likelihood estimate. Coordinates (x, y, z) are reported in Talairach convention; BA, Brodmann area; ALE, Activation likelihood estimate.

Supplementary References

Anderson, J. R., Lee, H. S., & Fincham, J. M. (2014). Discovering the structure of mathematical problem solving. *NeuroImage, 97*, 163-177.

Anderson, B., Soliman, S., O'Malley, S., Danckert, J., & Besner, D. (2015). Control over the strength of connections between modules: a double dissociation between stimulus format and task revealed by Granger causality mapping in fMRI. *Frontiers in Psychology*, *6*.

Andin, J., Fransson, P., Rönnberg, J., & Rudner, M. (2015). Phonology and arithmetic in the language–calculation network. *Brain and Language, 143*, 97-105.

Andres, M., Pelgrims, B., Michaux, N., Olivier, E., & Pesenti, M. (2011). Role of distinct parietal areas in arithmetic: an fMRI-guided TMS study. *Neuroimage*, *54*(4), 3048-3056.

Andres, M., Michaux, N., & Pesenti, M. (2012). Common substrate for mental arithmetic and finger representation in the parietal cortex. *NeuroImage, 62*(3), 1520-1528.

Ansari, D. (2007). Does the parietal cortex distinguish between "10,""ten," and ten dots?. *Neuron*, *53*(2), 165-167.

Ansari, D., & Dhital, B. (2006). Age-related changes in the activation of the intraparietal sulcus during nonsymbolic magnitude processing: An event-related functional magnetic resonance imaging study. *Journal of Cognitive Neuroscience, 18*(11), 1820-1828. doi: http://dx.doi.org/10.1162/jocn.2006.18.11.1820

Ansari. D., Garcia, N., Lucas, E., Hamon, K., & Dhital, B. (2005). Neural correlates of symbolic number processing in children and adults. *NeuroReport, 16*(16), 1769-1773.

Ansari, D., Grabner, R. H., Koschutnig, K., Reishofer, G., & Ebner, F. (2011). Individual differences in mathematical competence modulate brain responses to arithmetic errors: An fMRI study. *Learning and Individual Differences*, *21*(6), 636-643.

Audoin, B., Ibarrola, D., Duong, M. A., Pelletier, J., Confort-Gouny, S., Malikova, I., … & Ranjeva, J. P. (2005). Functional MRI study of PASAT in normal subjects. *Magnetic Resonance Materials in Physics, Biology and Medicine, 18*(2), 96-102.

Benn, Y., Webb, T. L., Chang, B. P., Sun, Y. H., Wilkinson, I. D., & Farrow, T. F. (2014). The neural basis of monitoring goal progress. *Frontiers in Huma n Neuroscience, 8*, 688.

Bulthé, J., De Smedt, B., & de Beeck, H. O. (2014). Format-dependent representations of symbolic and non-symbolic numbers in the human cortex as revealed by multi-voxel pattern analyses. *NeuroImage, 87*, 311-322.

Cantlon, J. F., Brannon, E. M., Carter, E. J., & Pelphrey, K. A. (2006). Functional imaging of numerical processing in adults and 4-y-old children. *PLoS Biology, 4*(5), 844-854.

Cavdaroglu, S., Katz, C., & Knops, A. (2015). Dissociating estimation from comparison and response eliminates parietal involvement in sequential numerosity perception. *NeuroImage*, *116*, 135-148.

Chen, C., Zhou, X., Chen, C., Dong, Q., Zang, Y., Qiao, S., ..., & Gong, Q. (2007). The neural basis of processing anomalous information. *NeuroReport*, *18*(8), 747-751.

Chiao, J. Y., Harada, T., Oby, E. R., Li, Z., Parrish, T., & Bridge, D. J. (2009). Neural representations of social status hierarchy in human inferior parietal cortex. *Neuropsychologia*, *47*(2), 354-363.

Chochon, F., Cohen, L., Van De Moortele, P. F., & Dehaene, S. (1999). Differential contributions of the left and right inferior parietal lobules to number processing. *Journal of Cognitive Neuroscience, 11*(6), 617-630.

Cohen-Kadosh, R., Henik, A., Rubinsten, O., Mohr, H., Dori, H., van de Ven, V., ..., & Linden, D. E. (2005). Are numbers special? the comparison systems of the human brain investigated by fMRI. *Neuropsychologia*, *43*(9), 1238-1248.

Cohne-Kadosh, R., Cohen-Kadosh, K., Linden, D. E., Gevers, W., Berger, A., & Henik, A. (2007). The brain locus of interaction between number and size: A combined functional magnetic resonance imaging and event-related potential study. *Journal of Cognitive Neuroscience*, *19*(6), 957-970.

Critchley, H. D., Tang, J., Glaser, D., Butterworth, B., & Dolan, R. J. (2005). Anterior cingulate activity during error and autonomic response. *NeuroImage*, *27*(4), 885-895.

Daniels, C., Witt, K., Wolff, S., Jansen, O., & Deuschl, G. (2003). Rate dependency of the human cortical network subserving executive functions during generation of

random number series–a functional magnetic resonance imaging study. *Neuroscience Letters*, *345*(1), 25-28.

Davis, N., Cannistraci, C. J., Rogers, B. P., Gatenby, J. C., Fuchs, L. S., Anderson, A. W., & Gore, J. C. (2009a). The neural correlates of calculation ability in children: an FMRI study. *Magnetic Resonance Imaging, 27*, 1187-1197.

Davis, N., Cannistraci, C. J., Rogers, B. P., Gatenby, C., Fuchs, L. S., Anderson, A. W., & Gore, J. C. (2009b). Aberrant functional activation in school age children at-risk for mathematical disability: A functional study of simple arithmetic skill. *Neuropsychologia, 47*, 2470-2479

Dehaene, S., Spelke, E., Pinel, P., Stanescu, R., & Tsivkin, S. (1999). Sources of mathematical thinking: Behavioral and brain-imaging evidence. *Science, 284*(5416), 970-974.

Delazer, M., Domahs, F., Bartha, L., Brenneis, C., Lochy, A., Trieb, T., & Benke, T. (2003). Learning complex arithmetic—an fMRI study. *Cognitive Brain Research, 18*(1), 76-88.

Delazer, M., Domahs, F., Lochy, A., Bartha, L., Christian, B., & Trieb, T. (2004). The acquisition of arithmetic knowledge-an FMRI study. *Cortex, 40*(1), 166-167.

Demeyere, N., Rotshtein, P., & Humphreys, G. W. (2014). Common and dissociated mechanisms for estimating large and small dot arrays: Value-specific fMRI adaptation. *Human Brain Mapping*, *35*(8), 3988-4001.

De Pisapia, N., Slomski, J. A., & Braver, T. S. (2007). Functional specializations in lateral prefrontal cortex associated with the integration and segregation of information in working memory. *Cerebral Cortex, 17*(5), 993-1006.

De Visscher, A., Berens, S. C., Keidel, J. L., Noël, M. P., & Bird, C. M. (2015). The interference effect in arithmetic fact solving: an fMRI study. *NeuroImage, 116*, 92-101.

Dormal, V., Andres, M., Dormal, G., & Pesenti, M. (2010). Mode-dependent and mode-independent representations of numerosity in the right intraparietal sulcus. *NeuroImage*, *52*(4), 1677-1686.

Eger, E., Sterzer, P., Russ, M. O., Giraud, A. L., & Kleinschmidt, A. (2003). A supramodal number representation in human intraparietal cortex. *Neuron*, *37*(4), 719-726.

Eickhoff, S. B., Bzdok, D., Laird, A. R., Kurth, F., & Fox, P. T. (2012). Activation likelihood estimation meta-analysis revisited. *NeuroImage*, *59*(3), 2349-2361.

Eickhoff, S. B., Laird, A. R., Fox, P. M., Lancaster, J. L., & Fox, P. T. (2017). Inplementation errors in the GingerALE Software: Description and recommendations. *Human Brain Mapping, 38*(1), 7-11.

Emerson, R. W., & Cantlon, J. F. (2015). Continuity and change in children's longitudinal neural responses to numbers. *Developmental Science*, *18*(2), 314-326.

Fehr, T., Code, C., & Herrmann, M. (2007). Common brain regions underlying different arithmetic operations as revealed by conjunct fMRI–BOLD activation. *Brain Research, 1172*, 93-102.

Feng, X., Peng, L., Chang-Quan, L., Yi, L., & Hong, L. (2014). Relational complexity modulates activity in the prefrontal cortex during numerical inductive reasoning: An fMRI study. *Biological Psychology, 101*, 61-68.

Franklin, M. S., & Jonides, J. (2009). Order and magnitude share a common representation in parietal cortex. *Journal of Cognitive Neuroscience*, *21*(11), 2114-2120.

Göbel, S. M., Johansen-Berg, H., Behrens, T., & Rushworth, M. F. (2004). Response-selection-related parietal activation during number comparison. *Journal of Cognitive Neuroscience*, *16*(9), 1536-1551.

Grabner, R. H., Ansari, D., Koschutnig, K., Reishofer, G., & Ebner, F. (2013). The function of the left angular gyrus in mental arithmetic: evidence from the associative confusion effect. *Human Brain Mapping, 34*(5), 1013-1024.

Grabner, R. H., Ischebeck, A., Reishofer, G., Koschutnig, K., Delazer, M., Ebner, F., & Neuper, C. (2009). Fact learning in complex arithmetic and figural‐spatial tasks: The role of the angular gyrus and its relation to mathematical competence. *Human Brain Mapping, 30*(9), 2936-2952.

Grabner, R. H., Saalbach, H., & Eckstein, D. (2012). Language‐Switching Costs in Bilingual Mathematics Learning. *Mind, Brain, and Education, 6*(3), 147-155.

Grabner, R. H., Ansari, D., Reishofer, G., Stern, E., Ebner, F., & Neuper, C. (2007). Individual differences in mathematical competence predict parietal brain activation during mental calculation. *Neuroimage*, *38*(2), 346-356.

Gullick, M. M., Sprute, L. A., & Temple, E. (2011). Individual differences in working memory, nonverbal IQ, and mathematics achievement and brain mechanisms associated with symbolic and nonsymbolic number processing. *Learning and Individual Differences*, *21*(6), 644-654.

Gullick, M. M., & Wolford, G. (2013). Understanding less than nothing: children's neural response to negative numbers shifts across age and accuracy. *Frontiers in Psychology, 4*, 1-17.

Gullick, M. M., & Wolford, G. (2014). Brain systems involved in arithmetic with positive versus negative numbers. *Human Brain Mapping, 35*(2), 539-551.

Hanakawa, T., Honda, M., Okada, T., Fukuyama, H., & Shibasaki, H. (2003). Differential activity in the premotor cortex subdivisions in humans during mental calculation and verbal rehearsal tasks: a functional magnetic resonance imaging study. *Neuroscience Letters, 347*(3), 199-201.

Harada, T., Bridge, D., & Chiao, J. Y. (2013). Dynamic social power modulates neural basis of math calculation. *Frontiers in Human Neuroscience, 6*, 350.

He, L., Zuo, Z., Chen, L., & Humphreys, G. (2014). Effects of number magnitude and notation at 7T: separating the neural response to small and large, symbolic and nonsymbolic number. *Cerebral Cortex*, *24*(8), 2199-2209.

Holloway, I. D., & Ansari, D. (2010). Developmental specialization in the right intraparietal sulcus for the abstract representation of numerical magnitude. *Journal of Cognitive Neuroscience*, *22*(11), 2627-2637.

Hsu, C. W., & Goh, J. O. (2016). Distinct and Overlapping Brain Areas Engaged during Value-Based, Mathematical, and Emotional Decision Processing. *Frontiers in Human Neuroscience*, *10*.

Hugdahl, K., Specht, K., Biringer, E., Weis, S., Elliott, R., Hammar, Å., … & Lund, A. (2007). Increased parietal and frontal activation after remission from recurrent major depression: a repeated fMRI study. *Cognitive Therapy and Research*, *31*(2), 147-160.

Hung, Y. H., Pallier, C., Dehaene, S., Lin, Y. C., Chang, A., Tzeng, O. J. L., & Wu, D. H. (2015). Neural correlates of merging number words. *NeuroImage*, *122*, 33-43.

Ischebeck, A., Zamarian, L., Egger, K., Schocke, M., & Delazer, M. (2007). Imaging early practice effects in arithmetic. *NeuroImage, 36*(3), 993-1003.

Ischebeck, A., Zamarian, L., Schocke, M & Delazer, M. (2009). Flexible transfer of knowledge in mental arithmetic — An fMRI study. *NeuroImage, 44*, 1103-1112.

Ischebeck, A., Zamarian, L., Siedentopf, C., Koppelstätter, F., Benke, T., Felber, S., & Delazer, M. (2006). How specifically do we learn? Imaging the learning of multiplication and subtraction. *NeuroImage*, *30*(4), 1365-1375.

Jost, K., Khader, P., Burke, M., Bien, S., & Rösler, F. (2009). Dissociating the solution processes of small, large, and zero multiplications by means of fMRI. *NeuroImage*, *46*(1), 308-318.

Kanjlia, S., Lane, C., Feigenson, L., & Bedny, M. (2016). Absence of visual experience modifies the neural basis of numerical thinking. *Proceedings of the National Academy of Sciences*, *113*(40), 11172-11177.

Kansaku, K., Carver, B., Johnson, A., Matsuda, K., Sadato, N., & Hallett, M. (2007). The role of the human ventral premotor cortex in counting successive stimuli. *Experimental Brain Research*, *178*(3), 339-350.

Kaufmann, L., Koppelstaetter, F., Delazer, M., Siedentopf, C., Rhomberg, P., Golaszewski, S., ..., & Ischebeck, A. (2005). Neural correlates of distance and congruity effects in a numerical Stroop task: an event-related fMRI study. *NeuroImage*, *25*(3), 888-898.

Kaufman, L., Stephan E. V., Wood, G., Kremser, C., Schocke, M., Zimmerhackl, LB., & Koten, J. W. (2008). A developmental fMRI study of nonsymbolic numerical and spatial processing. *Cortex, 44*, 376-385.

Kawashima, R., Taira, M., Okita, K., Inoue, K., Tajima, N., Yoshido, H., … & Fukuda, H. (2004). A functional MRI study of simple arithmetic—a comparison between children and adults. *Cognitive Brain Research, 18*, 225-231.

Keller, K., & Menon, V. (2009). Gender differences in the functional and structural neuroanatomy of mathematical cognition. *NeuroImage*, *47*(1), 342-352.

Klein, E., Willmes, K., Dressel, K., Domahs, F., Wood, G., Nuerk, H. C., & Moeller, K. (2010). Categorical and continuous-disentangling the neural correlates of the carry effect in multi-digit addition. *Behavioral and Brain Functions*, *6*(1), 70.

Kong, J., Wang, C., Kwong, K., Vangel, M., Chua, E., & Gollub, R. (2005). The neural substrate of arithmetic operations and procedure complexity. *Cognitive Brain Research*, *22*(3), 397-405.

Krueger, F., Spampinato, M. V., Pardini, M., Pajevic, S., Wood, J. N., Weiss, G. H., … & Grafman, J. (2008). Integral calculus problem solving: an fMRI investigation. *NeuroReport*, *19*(11), 1095.

Kuo, B. C., Yeh, Y. Y., Chen, D. Y., Liang, K. C., & Chen, J. H. (2008). The capacity constraint in the prefrontal and parietal regions for coordinating dual arithmetic tasks. *Brain Research*, *1199*, 100-110.

Landrø, N. I., Rund, B. R., Lund, A., Sundet, K., Mjellem, N., Asbjørnsen, A., ..., & Egeland, J. (2001). Honig's model of working memory and brain activation: an fMRI study. *NeuroReport*, *12*(18), 4047-4054.

Le Clec'H, G., Dehaene, S., Cohen, L., Mehler, J., Dupoux, E., Poline, J. B., ..., & Le Bihan, D. (2000). Distinct cortical areas for names of numbers and body parts independent of language and input modality. *NeuroImage*, *12*(4), 381-391.

Lee, K. M. (2000). Cortical areas differentially involved in multiplication and subtraction: a functional magnetic resonance imaging study and correlation with a case of selective acalculia. *Annals of Neurology*, *48*(4), 657-661.

Leibovich, T., Henik, A., & Salti, M. (2015). Numerosity processing is context driven even in the subitizing range: An fMRI study. *Neuropsychologia*, *77*, 137-147.

Leibovich, T., Vogel, S. E., Henik, A., & Ansari, D. (2015). Asymmetric processing of numerical and nonnumerical magnitudes in the brain: an fMRI study. *Journal of cognitive neuroscience, 28 (1)*, 166-167.

Leroux, G., Spiess, J., Zago, L., Rossi, S., Lubin, A., Turbelin, M. R., ... & Joliot, M. (2009). Adult brains don't fully overcome biases that lead to incorrect performance during cognitive development: an fMRI study in young adults completing a Piaget-like task. *Developmental science*, *12*(2), 326-338.

Liang, P., Jia, X., Taatgen, N. A., Borst, J. P., & Li, K. (2016). Activity in the fronto-parietal network indicates numerical inductive reasoning beyond calculation: An fMRI study combined with a cognitive model. *Scientific reports*, *6*.

Liu, J., Zhang, H., Chen, C., Chen, H., Cui, J., & Zhou, X. (2017). The neural circuits for arithmetic principles. *NeuroImage*, *147*, 432-446.

Liu, X., Wang, H., Corbly, C. R., Zhang, J., & Joseph, J. E. (2006). The involvement of the inferior parietal cortex in the numerical Stroop effect and the distance effect in a

two-digit number comparison task. *Journal of Cognitive Neuroscience*, *18*(9), 1518-1530.

Menon, V., Rivera, S. M., White, C. D., Glover, G. H., & Reiss, A. L. (2000). Dissociating prefrontal and parietal cortex activation during arithmetic processing. *NeuroImage*, *12*(4), 357-365.

Moher, D., Liberati, A., Tetzlaff, J., Altman, D. G., & Prisma Group. (2009). Preferred reporting items for systematic reviews and meta-analyses: the PRISMA statement. *PLoS med*, *6*(7), e1000097.

Molko, N., Cachia, A., Rivière, D., Mangin, J. F., Bruandet, M., Le Bihan, D., ..., & Dehaene, S. (2003). Functional and structural alterations of the intraparietal sulcus in a developmental dyscalculia of genetic origin. *Neuron*, *40*(4), 847-858.

Mondt, K., Struys, E., Van den Noort, M., Balériaux, D., Metens, T., Paquier, P., … & Denolin, V. (2011). Neural differences in bilingual children's arithmetic processing depending on language of instruction. *Mind, Brain and Education, 5*(2), 79-88.

Newman, S. D., Willoughby, G., & Pruce, B. (2011). The effect of problem structure on problem-solving: an fMRI study of word versus number problems. *Brain Research*, *1410*, 77-88.

Ogata, Y., Horaguchi, T., Watanabe, N., & Yamamoto, M. (2011). Comparison of the choice effect and the distance effect in a number-comparison task by FMRI. *PloS One*, *6*(6), e21716.

Piazza, M., Izard, V., Pinel, P., Le Bihan, D., & Dehaene, S. (2004). Tuning curves for approximate numerosity in the human intraparietal sulcus. *Neuron*, *44*(3), 547-555.

Piazza, M., Mechelli, A., Price, C. J., & Butterworth, B. (2006). Exact and approximate judgements of visual and auditory numerosity: An fMRI study. *Brain Research*, *1106*(1), 177-188.

Piazza, M., Pinel, P., Le Bihan, D., & Dehaene, S. (2007). A magnitude code common to numerosities and number symbols in human intraparietal cortex. *Neuron*, *53*(2), 293-305.

Pinel, P., & Dehaene, S. (2013). Genetic and environmental contributions to brain activation during calculation. *Neuroimage*, *81*, 306-316.

Pinel, P., Dehaene, S., Riviere, D., & LeBihan, D. (2001). Modulation of parietal activation by semantic distance in a number comparison task. *NeuroImage*, *14*(5), 1013-1026.

Pinel, P., Le Clec'H, G., Van de Moortele, P. F., Naccache, L., Le Bihan, D., & Dehaene, S. (1999). Event-related fMRI analysis of the cerebral circuit for number comparison. *NeuroReport*, *10*(7), 1473-1479.

Pinel, P., Piazza, M., Le Bihan, D., & Dehaene, S. (2004). Distributed and overlapping cerebral representations of number, size, and luminance during comparative judgments. *Neuron*, *41*(6), 983-993.

Pletzer, B. (2016). Sex differences in number processing: Differential systems for subtraction and multiplication were confirmed in men, but not in women. *Scientific Reports*, *6*.

Prabhakaran, V., Rypma, B., & Gabrieli, J. D. (2001). Neural substrates of mathematical reasoning: A functional magnetic resonance imaging study of neocortical activation during performance of the necessary arithmetic operations test. *Neuropsychology*, *15*(1), 115.

Prado, J., Mutreja, R., Zhang, H., Mehta, R., Desroches, A. S., Minas, J. E., & Booth, J. R. (2011). Distinct representations of subtraction and multiplication in the neural systems for numerosity and language. *Human Brain Mapping*, *32*(11), 1932-1947.

Rickard, T. C., Romero, S. G., Basso, G., Wharton, C., Flitman, S., & Grafman, J. (2000). The calculating brain: an fMRI study. *Neuropsychologia*, *38*(3), 325-335.

Robertson, B. D., Hiebert, N. M., Seergobin, K. N., Owen, A. M., & MacDonald, P. A. (2015). Dorsal striatum mediates cognitive control, not cognitive effort per se, in decision-making: An event-related fMRI study. *NeuroImage*, *114*, 170-184.

Salimpoor, V. N., Chang, C., & Menon, V. (2010). Neural basis of repetition priming during mathematical cognition: Repetition suppression or repetition enhancement? *Journal of Cognitive Neuroscience*, *22*(4), 790-805.

Sammer, G., Blecker, C., Gebhardt, H., Bischoff, M., Stark, R., Morgen, K., & Vaitl, D. (2007). Relationship between regional hemodynamic activity and simultaneously recorded EEG-theta associated with mental arithmetic-induced workload. *Human Brain Mapping*, *28*(8), 793-803.

Simon, O., Mangin, J. F., Cohen, L., Le Bihan, D., & Dehaene, S. (2002). Topographical layout of hand, eye, calculation, and language-related areas in the human parietal lobe. *Neuron*, *33*(3), 475-487.

Soylu, F., & Newman, S. D. (2016). Anatomically ordered tapping interferes more with one-digit addition than two-digit addition: a dual-task fMRI study. *Cognitive processing*, *17*(1), 67-77.

Skagerlund, K., Karlsson, T., & Träff, U. (2016). Magnitude processing in the brain: an fMRI study of time, space, and numerosity as a shared cortical system. *Frontiers in human neuroscience*, *10*.

Stanescu-Cosson, R., Pinel, P., van de Moortele, P. F., Le Bihan, D., Cohen, L., & Dehaene, S. (2000). Understanding dissociations in dyscalculia. *Brain*, *123*(11), 2240-2255.

Sokolowski, H. M., Fias, W., Mousa, A., & Ansari, D. (2017). Common and distinct brain regions in both parietal and frontal cortex support symbolic and nonsymbolic number processing in humans: A functional neuroimaging meta-analysis. *NeuroImage, 146*, 376-394.

Taillan, J., Ardiale, E., Anton, J. L., Nazarian, B., Félician, O., & Lemaire, P. (2015). Processes in arithmetic strategy selection: A fMRI study. *Frontiers in Psychology*, *6*.

Tan, H. Y., Chen, Q., Goldberg, T. E., Mattay, V. S., Meyer-Lindenberg, A., Weinberger, D. R., & Callicott, J. H. (2007). Catechol-O-methyltransferase Val158Met modulation of prefrontal–parietal–striatal brain systems during arithmetic and temporal transformations in working memory. *Journal of Neuroscience*, *27*(49), 13393-13401.

Tang, J., Critchley, H. D., Glaser, D. E., Dolan, R. J., & Butterworth, B. (2006). Imaging informational conflict: A functional magnetic resonance imaging study of numerical Stroop. *Journal of Cognitive Neuroscience*, *18*(12), 2049-2062.

Tenison, C., Fincham, J. M., & Anderson, J. R. (2014). Detecting math problem solving strategies: An investigation into the use of retrospective self-reports, latency and fMRI data. *Neuropsychologia*, *54*, 41-52.

Tschentscher, N., & Hauk, O. (2014). How are things adding up? Neural differences between arithmetic operations are due to general problem solving strategies. *NeuroImage*, *92*, 369-380.

Tschentscher, N., Hauk, O., Fischer, M. H., & Pulvermüller, F. (2012). You can count on the motor cortex: finger counting habits modulate motor cortex activation evoked by numbers. *NeuroImage*, *59*(4), 3139-3148.

Turkeltaub, P. E., Eickhoff, S. B., Laird, A. R., Fox, M., Wiener, M., & Fox, P. (2012). Minimizing within-experiment and within-group effects in activation likelihood estimation meta-analyses. *Human brain mapping, 33*(1), 1-13.

Ulrich, M., Keller, J., & Grön, G. (2016). Neural signatures of experimentally induced flow experiences identified in a typical fMRI block design with BOLD imaging. *Social cognitive and affective neuroscience*, *11*(3), 496-507.

Vassena, E., Silvetti, M., Boehler, C. N., Achten, E., Fias, W., & Verguts, T. (2014). Overlapping neural systems represent cognitive effort and reward anticipation. *PLoS One*, *9*(3), e91008.

Venkatraman, V., Siong, S. C., Chee, M. W., & Ansari, D. (2006). Effect of language switching on arithmetic: A bilingual fMRI study. *Journal of Cognitive Neuroscience*, *18*(1), 64-74.

Vogel, S. E., Grabner, R. H., Schneider, M., Siegler, R. S., & Ansari, D. (2013). Overlapping and distinct brain regions involved in estimating the spatial position of numerical and non-numerical magnitudes: an fMRI study. *Neuropsychologia*, *51*(5), 979-989.

Wang, Y., Lin, L., Kuhl, P., & Hirsch, J. (2007). Mathematical and linguistic processing differs between native and second languages: An fMRI study. *Brain Imaging and Behavior*, *1*(3-4), 68-82.

Wang, L. Q., Wang, M. S., & Saito, M. (2005). Functional neuroanatomy involved in automatic order mental arithmetic and recitation of the multiplication table: A concurrent quantity processing mechanism in actual computation. *Electrical Engineering in Japan*, *153*(2), 39-44.

Wessa, M., Heissler, J., Schönfelder, S., & Kanske, P. (2013). Goal-directed behavior under emotional distraction is preserved by enhanced task-specific activation. *Social Cognitive and Affective Neuroscience*, nsr098.

Wood, G., Nuerk, H. C., Sturm, D., & Willmes, K. (2008). Using parametric regressors to disentangle properties of multi-feature processes. *Behavioral and Brain Functions*, *4*(1), 38.

Wood, G., Nuerk, H. C., & Willmes, K. (2006). Neural representations of two-digit numbers: A parametric fMRI study. *NeuroImage*, *29*(2), 358-367.

Wu, S. S., Chang, T. T., Majid, A., Caspers, S., Eickhoff, S. B., & Menon, V. (2009). Functional heterogeneity of inferior parietal cortex during mathematical cognition

assessed with cytoarchitectonic probability maps. *Cerebral Cortex*, *19*(12), 2930-2945.

Yang, Y., Zhong, N., Imamura, K., & Lei, X. (2013). Common and Dissociable Neural Substrates for 2-Digit Simple Addition and Subtraction. In *International Conference on Brain and Health Informatics* (pp. 92-102). Springer International Publishing.

Yi-Rong, N., Si-Yun, S., Zhou-Yi, G., Si-Run, L., Yun, B., Song-Hao, L., & Chan, W. Y. (2011). Dissociated brain organization for two-digit addition and subtraction: An fMRI investigation. *Brain Research Bulletin*, *86*(5), 395-402.

Zago, L., Petit, L., Turbelin, M. R., Andersson, F., Vigneau, M., & Tzourio-Mazoyer, N. (2008). How verbal and spatial manipulation networks contribute to calculation: an fMRI study. *Neuropsychologia*, *46*(9), 2403-2414.

Zarnhofer, S., Braunstein, V., Ebner, F., Koschutnig, K., Neuper, C., Reishofer, G., & Ischebeck, A. (2012). The influence of verbalization on the pattern of cortical activation during mental arithmetic. *Behavioral and Brain Functions*, *8*(1), 13.

Zhou, X., Chen, C., Zang, Y., Dong, Q., Chen, C., Qiao, S., & Gong, Q. (2007). Dissociated brain organization for single-digit addition and multiplication. *NeuroImage*, *35*(2), 871-880.

Zhou, X., Chen, C., Zhang, H., Xue, G., Dong, Q., Jin, Z., ..., & Jiang, T. (2006). Neural substrates for forward and backward recitation of numbers and the alphabet: A close examination of the role of intraparietal sulcus and perisylvian areas. *Brain Research*, *1099*(1), 109-120.
